# GADD45A and CDKN1A are involved in apoptosis and cell cycle modulatory effects of viscumTT with further inactivation of the STAT3 pathway

**DOI:** 10.1038/s41598-018-24075-x

**Published:** 2018-04-10

**Authors:** Susann Kleinsimon, Enya Longmuss, Jana Rolff, Sebastian Jäger, Angelika Eggert, Catharina Delebinski, Georg Seifert

**Affiliations:** 1 Department of Pediatric Oncology/Hematology, Otto-Heubner-Centre for Pediatric and Adolescent Medicine (OHC), Charité, Universitätsmedizin, Berlin, Germany; 2 Institute of Pharmacy, Department of Biology, Chemistry, Pharmacy, Freie Universität, Berlin, Germany; 3 Institute of Molecular Bioscience, The University of Queensland, St. Lucia Qld, Australia; 4 EPO GmbH, Experimental Pharmacology & Oncology, Berlin, Germany; 5 Birken AG, Niefern-Öschelbronn, Germany

## Abstract

ViscumTT, a whole mistletoe preparation, has shown synergistic induction of apoptosis in several pediatric tumor entities. High therapeutic potential has previously been observed in Ewing’s sarcoma, rhabdomyosarcoma, ALL and AML. In this study, we analyzed modulatory effects on the cell cycle by viscumTT in three osteosarcoma cell lines with various *TP53* statuses. ViscumTT treatment induced G1 arrest in *TP53* wild-type and null-mutant cells, but S arrest in *TP53* mutant cells. Blockage of G1/S transition was accompanied by down-regulation of the key regulators CDK4, CCND1, CDK2, CCNE, CCNA. However, investigations on the transcriptional level revealed secondary TP53 participation. Cell cycle arrest was predominantly mediated by transcriptionally increased expression of *GADD45A* and *CDKN1A* and decreased *SKP2* levels. Enhanced *CDKN1A* and *GADD45A* expression further played a role in viscumTT-induced apoptosis with involvement of stress-induced MAPK8 and inactivation of MAPK1/3. Furthermore, viscumTT inhibited the pro-survival pathway STAT3 by dephosphorylation of the two sites, Tyr705 and Ser727, by down-regulation of total STAT3 and its direct downstream targets BIRC5 and C-MYC. Moreover, tests of the efficacy of viscumTT *in vivo* showing reduction of tumor volume confirmed the high therapeutic potential as an anti-tumoral agent for osteosarcoma.

## Introduction

ViscumTT is a whole mistletoe extract resulted from combination of two single extracts (viscum and TT). Viscum represents the aqueous part and is similar to conventional mistletoe preparations. It contains mainly hydrophilic mistletoe lectin I (ML I) as well as viscotoxins, whereby ML I is the main active constitutes and functioned as marker substance^1,2^. ML I is a glycoprotein belonging to the ribosome-inactivating proteins (RIP) type II and consists of a binding (B) and an activity (A) chain. The B chain binds to D-galactose at the cell surface and the A chain mediates its enzymatic activity in the cell^3,4^. TT represents the lipophilic part of viscumTT and contains mainly oleanolic- (OA) and betulinic acid (BA). Both are nearly water-insoluble and were solubilized with 2-hydroxypropyl-ß-cyclodextrin for application in watery environment^5^. OA is distinctly higher concentrated in the TT extract and is used as marker substance. For both main active constituents of viscumTT, ML I and OA, various anti-tumoral properties such as induction of apoptosis, cell cycle arrest and immunomodulatory functions have been described^6–11^. In previous studies, we have shown synergistic effects *in vitro* as well as high therapeutic effectiveness *in vivo* in a panel of tumor entities when both single extracts were combined (viscumTT)^12–18^.

The basic mechanism of action of viscumTT is not fully understood. Sequence analysis and proteomic profiling of viscumTT-treated Ewing’s sarcoma cells have provided information about the activated pathways^19^. ViscumTT and its single extracts viscum and TT are involved in activation of the stress-mediated mitogen-activated kinase (MAPK8) pathway, oxidative stress and Toll like receptor signaling^19^. European and Korean mistletoe mediated induction of apoptosis via activation of the phosphatidylinositol 3/protein kinase B (PI3K/AKT) pathway^20^ and mitogen-activated protein kinase 8/14 (MAPK8/MAPK14)^21^ signaling. Down-regulation of inhibitor of apoptosis proteins (IAPs) such as baculoviral inhibitor of apoptosis repeat-containing 5 (BIRC5, survivin) and X-linked inhibitor of apoptosis protein (XIAP) was observed in Ewing’s sarcoma, osteosarcoma and rhabdomyosarcoma cell lines^12–14^. Signal transducer and activator of transcription 3 (STAT3) which is often constitutively activated in diverse tumors^22^ was inhibited by a synthetic oleanolic derivative in multi-drug resistant osteosarcoma cells^23^ as well as by a fermented mistletoe preparation in gliomas^24^.

Tumor protein 53 (*TP53*) is one of the most mutated genes in human cancers^25^. Encoded tumor suppressor protein functions as key regulator of many cell processes including cell cycle regulation, senescence, survival, proliferation and apoptosis^26^. Around 10-20% of osteosarcomas have rearranged *TP53* and dysregulated cell cycles^27^. In healthy cells, TP53 is immediately induced and contributes to transcriptional activation of a range of target genes, e.g. BCL2-associated X (*BAX)*^28^, growth arrest and DNA damage inducible alpha (*GADD45A)*^29^, cyclin-dependent kinase inhibitor 1 A (*CDKN1A)*^30^ and *FAS*^31^, after cellular stress and DNA damage. In many cases, TP53 is not strictly required for drug-induced cell death and its contribution depends on dose, tissue and mutational background of the tumor^32^. Today, influencing cell cycle regulatory proteins may offer a promising approach in cancer therapy. Therefore, positive (cyclins, CCNs) and negative (cyclin-dependent kinase inhibitors, CDKNs) regulators of cyclin-dependent kinases (CDKs) are also of interest^33^.

Our present study reveals new insights into the mechanism of action of viscumTT regarding alteration of cell cycle progression and induction of apoptosis in osteosarcoma *in vitro*. We report the involvement of GADD45A and CDKN1A and show that STAT3 plays a role in molecular events. We also demonstrate high therapeutic efficacy of viscumTT in an osteosarcoma xenograft model.

## Results

### Viscum, TT and viscumTT inhibit cell cycle progression

**Figure 1:**
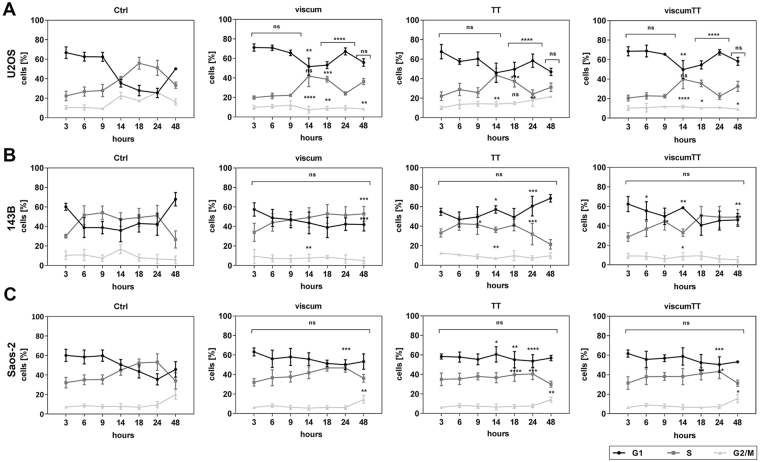
Viscum, TT and viscumTT have cell cycle modulatory effects. U2OS (**A**), 143B (**B**) and Saos-2 cells (**C**) were analyzed regarding to cell cycle distribution after viscum, TT and viscumTT treatment at different time points. Ethanol-fixed cells were stained by propidium iodide and analyzed by FACS. For viscum, mistletoe lectin (ML) 10 ng/mL, for TT, oleanolic acid (OA) 60 µg/mL and for viscumTT, ML+OA 5 ng/mL + 50 µg/mL were used. The means ± SD of cells in % in cell cycle phase were displayed in comparison to ctrl (n ≥ 3). Significant results are indicated as *p ≤ 0.05, **p ≤ 0.01, ***p ≤ 0.001, ****p ≤ 0.0001 related to untreated control (ctrl). Each experiment was independently performed thrice (n ≥ 3).

Synchronized osteosarcoma cell lines were treated with viscum, TT and viscumTT, and cell cycle distribution was analyzed at different time points. Viscum significantly arrested *TP53* wild-type (U2OS, Fig. 1A) and null-mutant (Saos-2) cells in G1 phase, whereas *TP53* mutant cells (143B) remained in S phase (Fig. 1B). On the other hand, TT led to G1 arrest in all cell lines. Likewise, viscumTT affected the cell cycle in *TP53* wild-type and null-mutant cells in G1 phase, whereas *TP53* mutant cells showed higher cell counts in S phase. In U2OS cells after TT and in Saos-2 cells after all treatments an increase in the number of cells in G2/M phase after 48 h was observed, indicating that complete stagnation had not occurred. The results of all treatments point to a cell cycle inhibitory effect by viscumTT in each cell line.

### Viscum, TT and viscumTT down-regulate CDKs and CCNs

**Figure 2:**
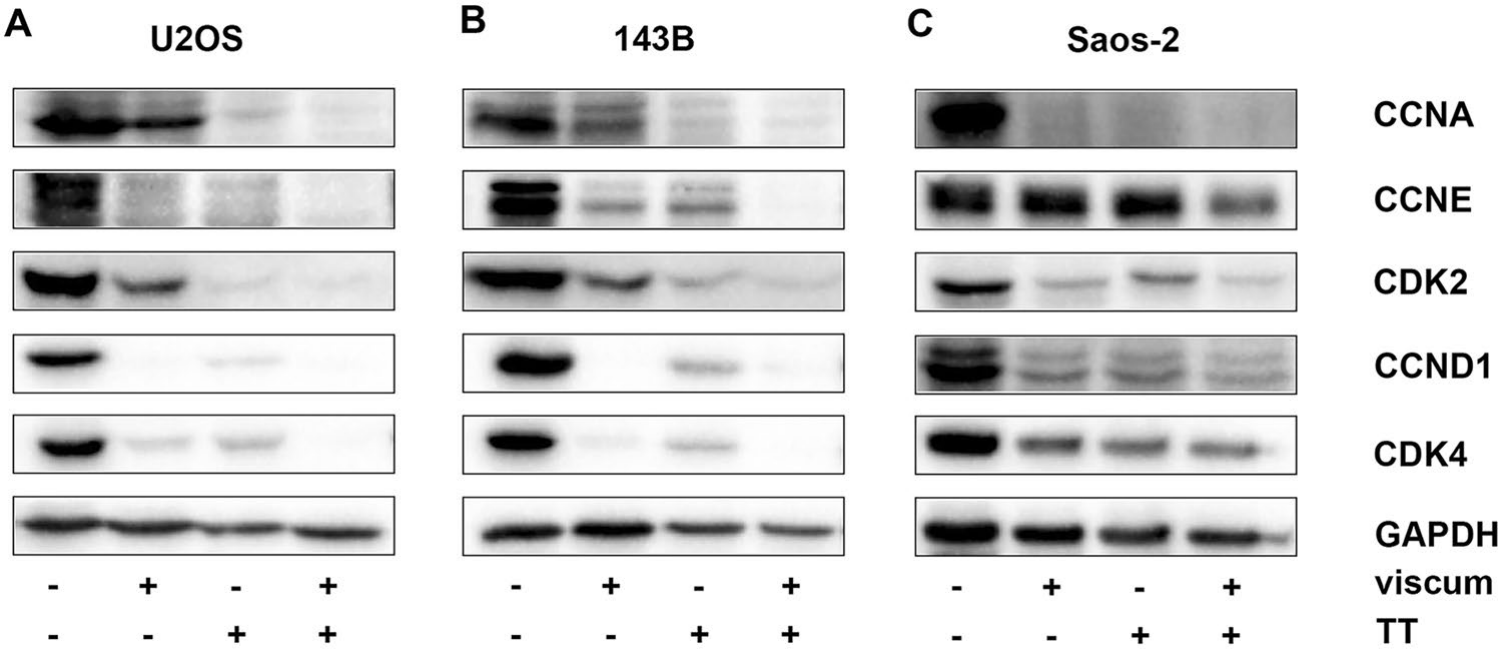
Key regulators of G1/S transition are down-regulated by each treatment. Whole cell lysates were analyzed by western blotting after 24 h of treatment and protein expression of cyclin A (CCNA), cyclin E (CCNE), cyclin-dependent kinase 2 (CDK2), cyclin D1 CCND1) and cyclin-dependent kinase 4 (CDK4) was detected. Cropped blots display representative images from three independent experiments of U2OS (**A**), 143B (**B**) and Saos-2 (**C**) cells after viscum (ML) 10 ng/mL, TT, oleanolic acid (OA) 60 µg/mL and viscumTT, ML+OA 5 ng/mL + 50 µg/mL treatment. ML was used as marker substance for viscum and OA for TT. GAPDH was used as loading control.

To investigate the observed cell cycle arrests, the key regulators of G1/S transition, CDK4 and cyclin D1 (CCND1), as well as CDK2 and CCNE/A protein levels were analyzed by western blotting after 24 h. CDK4 and CCND1 were strongly down-regulated after viscum, TT and viscumTT treatment in all three cell lines (Fig. 2A–C). CDK2, CCNE and CCNA were similar down-regulated in U2OS cells, but lower in 143B cells. CCNE expression was not affected in Saos-2 cells after viscum and TT treatment. The observed results suggest a direct effect on cell cycle regulatory proteins and confirm prevention of G1/S phase progression.

### Viscum, TT and viscumTT alter expression pattern of cell cycle-related genes

**Figure 3:**
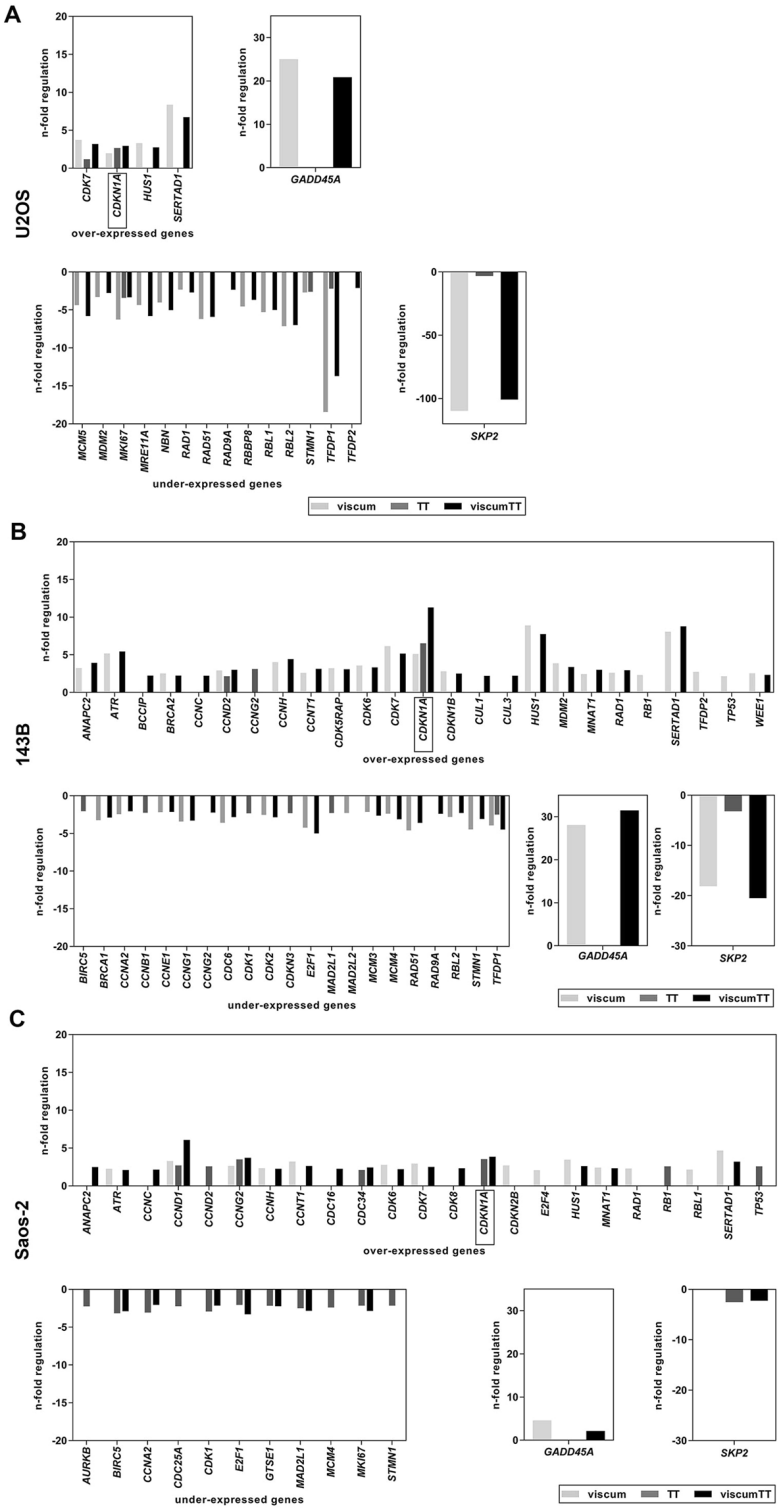
Viscum, TT and viscumTT alter cell cycle-related genes. Expression of cell cycle-related genes of U2OS (**A**), 143B (**B**), Saos-2 (**C**) cells were analyzed by RT² Profiler™ PCR Array after 24 h of viscum, TT and viscumTT treatment. For viscum, mistletoe lectin I (ML) 10 ng/mL, for TT, oleanolic acid (OA) 60 µg/mL and for viscumTT, ML+OA 5 ng/mL + 50 µg/mL were used. Array was performed once for each cell line and the fold-regulation cut-off was set to 2 and the p-value cut off was set to p ≤ 0.05 by the software.

For a first overview, direct effects on cell cycle regulatory target genes were analyzed using RT² Profiler™ PCR Array Human Cell Cycle after 24 h of treatment. The gene expression patterns of all cell lines showed alterations. Figure 3A–C show up- and down-regulated genes starting from 2-fold regulation to control as cut-off for significant change. After matching the results, five genes were found that were up-regulated and one gene which were down-regulated in each cell line after viscumTT treatment. One of these five up-regulated genes was *GADD45A* which showed a 30-fold increase in *TP53* wild-type and mutant cells after viscum and viscumTT treatment, but less in null mutant cells. CDKN1A a second up-regulated gene was triggered predominantly by TT and viscumTT treatment and to a distinctly greater extent in *TP53* mutant and null-mutant cells. *SKP2* as only down-regulated gene in all three cell lines showed approximately 70-fold decrease in *TP53* wild-type (U2OS) and around 20-fold decrease in mutant cells (143B) after viscum and viscumTT incubation. Null-mutant cells (Saos-2) showed only a weak change. Viscum, TT and viscumTT altered the transcriptional level of several cell cycle genes. Two of five up-regulated genes (*GADD45A*, *CDKN1A*) and *SKP2* as only down-regulated gene with the highest fold change were analyzed further. All fold changes of altered cell cycle genes are listed in supplementary information including clustergram to each cell line (Table S1A-S1C, Figure S1A–S1C).

### CDKN1A, GADD45A and SKP2 are transcriptionally affected by viscum, TT and viscumTT

**Figure 4:**
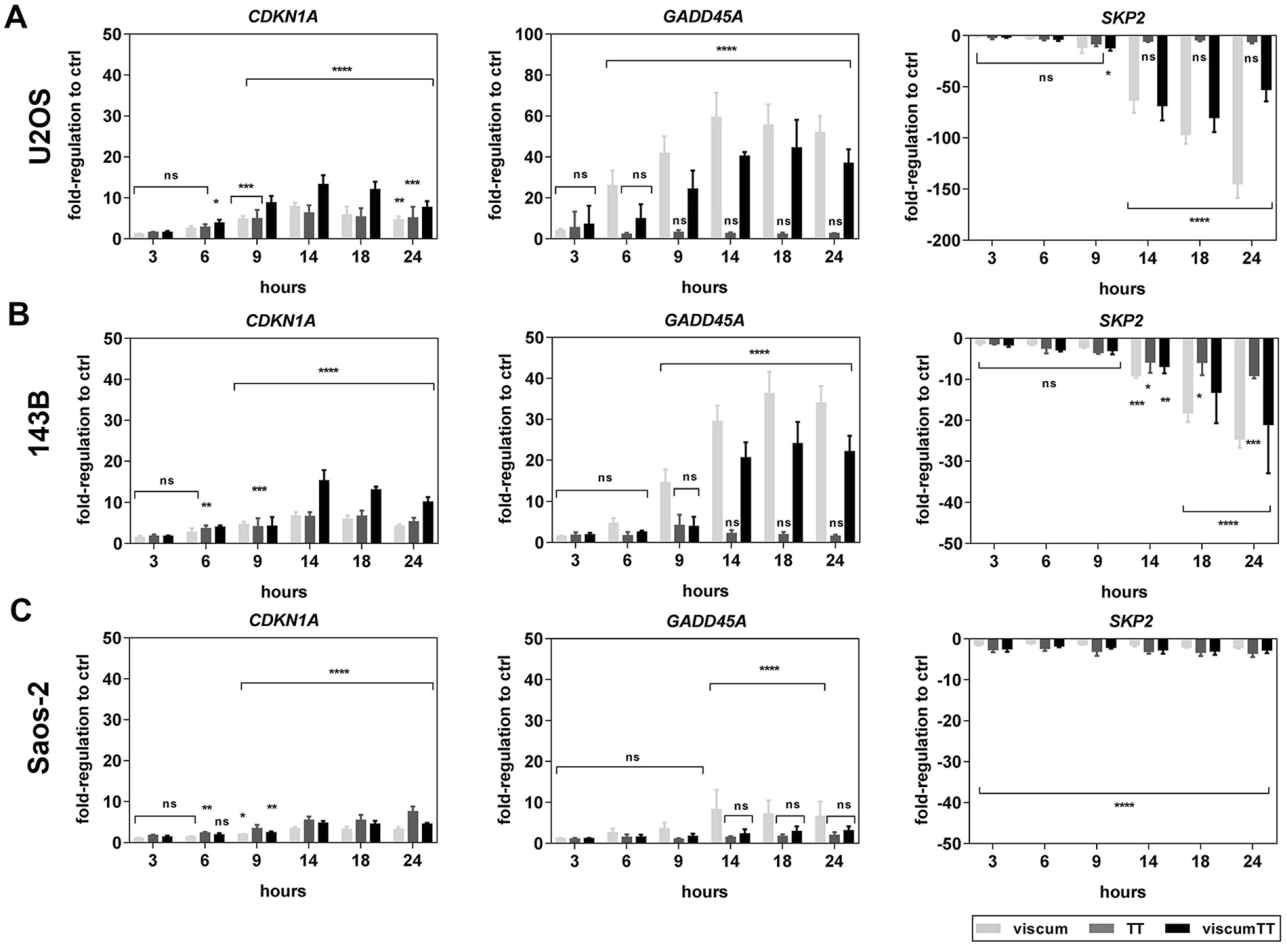
Cell cycle alterations based on up-regulation of CDKN1A and GADD45A and down-regulation of SKP2. Gene expression of CDKN1A, GADD45A and SKP2 was detected between 3 and 24 h. U2OS (**A**), 143B (**B**) and Saos-2 (**C**) cells were treated either with viscum, TT or viscumTT. The means of n-fold regulation ± SD related to untreated control (*p ≤ 0.05, **p ≤ 0.01, ***p ≤ 0.001, ****p ≤ 0.0001) are shown. Each experiment was independently performed thrice (n ≥ 3).

In order to validate the RT² Profiler™ PCR Array results, *CDKN1A*, *GADD45A* and *SKP2* expression was examined over the course of time using RT-qPCR. Viscum and viscumTT increased *CDKN1A* to a greater extent in *TP53* wild-type (up to 8-fold and 13-fold increase, respectively) and mutant cells (up to 7-fold and 15-fold increase, respectively), whereas TT (up to 8-fold increase) had the same effect in both cell lines. (Fig. 4A–C). Significant up-regulation started 6 h after treatment, peaked at 14 h for viscum/viscumTT in all cell lines and at 24 h for TT in Saos-2 cells. Likewise, GADD45A was periodically induced in each cell line but predominantly expressed by viscum and viscumTT, although the level was lower after viscumTT treatment. However, *GADD45A* was more strongly up-regulated in *TP53* wild-type (up to 56-fold after viscum and 44-fold after viscumTT) and mutant cells (up to 36-fold after viscum and 24-fold after viscumTT) than in null-mutant cells (Fig. 4A–C). TT did not influence the GADD45A level in any of the three cell lines. On the other hand, *SKP2* was drastically down-regulated (up to 140-fold decrease) in *TP53* wild type (U2OS) and mutant (143B) cells after viscum and viscumTT treatment (Fig. 4A,B), whereas TT led to an 8-fold decrease (Fig. 4A,B). In *TP53* null-mutant (Saos-2) cells, the effect was weaker (approximately 3-fold decrease, Fig. 4C) after all treatments. The results confirm *CDKN1A*, *GADD45A* and *SKP2* as transcriptional targets for viscum and viscumTT, while TT mainly influenced the expression of *CDKN1A* but not of *GADD45A*.

### Viscum, TT and viscumTT influence CDKN1A, GADD45A and SKP2 protein expression

**Figure 5:**
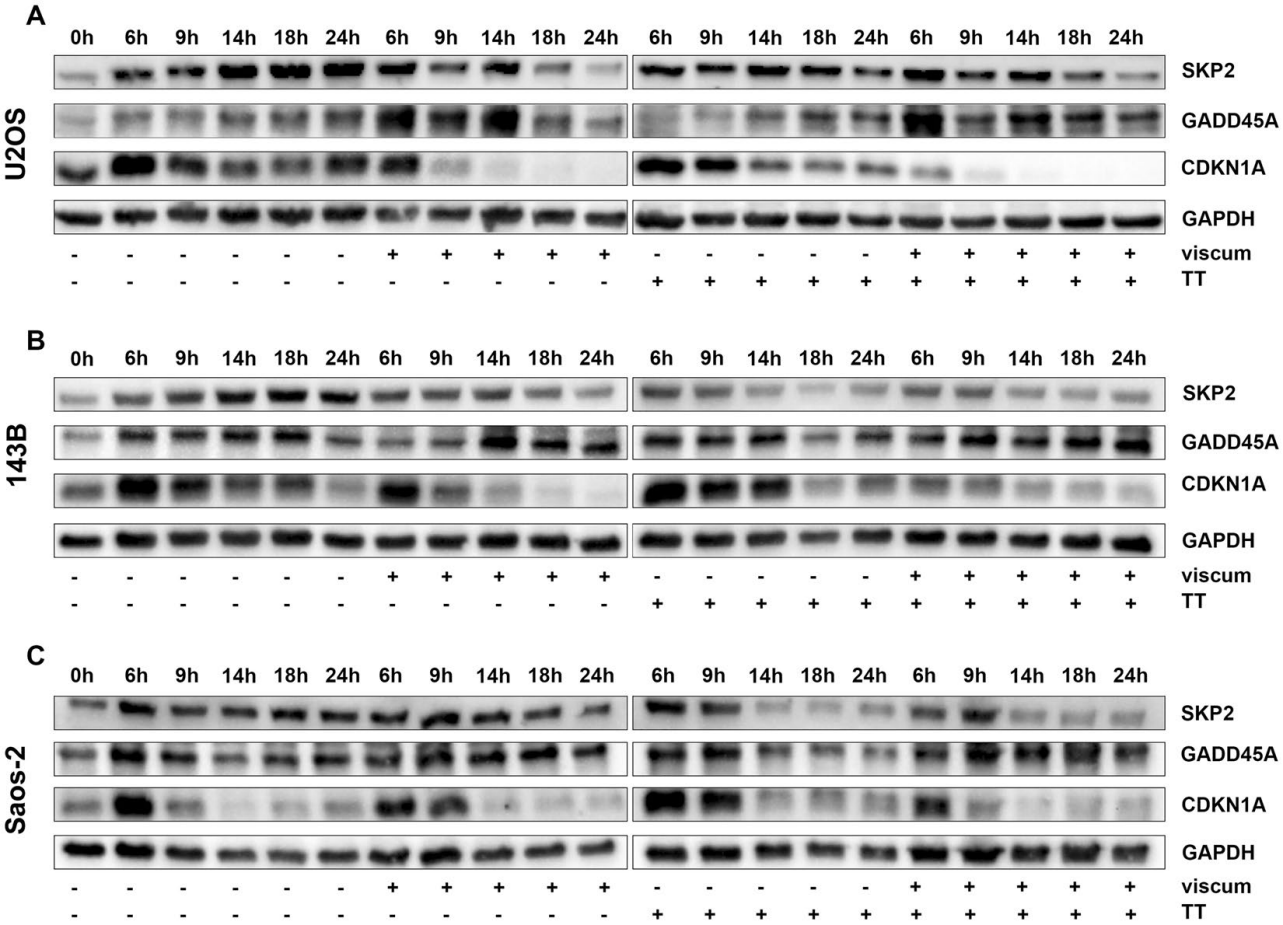
Viscum, TT and viscumTT affect GADD45A, CDKN1A and SKP2 on protein level. Blots demonstrate CDKN1A, GADD45A and SKP2 expression of whole cell lysates from U2OS (**A**), 143B (**B**) and Saos-2 (**C**) after different time points after viscum, TT and viscumTT treatment. GAPDH was used as loading control. Cropped blots display representative images from three independent experiments.

After investigation of gene expression, CDKN1A, GADD45A and SKP2 protein levels were analyzed by western blotting over the course of time. Viscum and viscumTT led to down-regulation of CDKN1A in *TP53* wild-type (U2OS) and mutant (143B) cells after 6/9 h of incubation, whereas null-mutant cells showed similar protein expression to control cells (Fig. 5A–C). TT initiated down-regulation of CDKN1A after 14 (U2OS) or 18 h (143B). In Saos-2 cells CDKN1A was higher in comparison to control after 9 h. GADD45A was distinctly higher expressed in U2OS cells after 6-14 h of viscum and viscumTT incubation. 143B cells showed stronger induction at 24 h in comparison to control after both treatments, whereas in Saos-2 cells a prolonged increase after starting at 14/18 h was seen. TT did not affect GADD45A in any cell line. SKP2 was distinctly down-regulated after viscum and viscumTT in U2OS, after viscum, TT and viscumTT in 143B and after TT and viscumTT in Saos-2 cells (Fig. 5A–C). Taken together, a divergence between gene and protein expression was observed in the case of CDKN1A whereas GADD45A and SKP2 protein correlated with the transcriptional level.

### Knock down of CDKN1A and GADD45A attenuates viscum, TT and viscumTT effect

**Figure 6:**
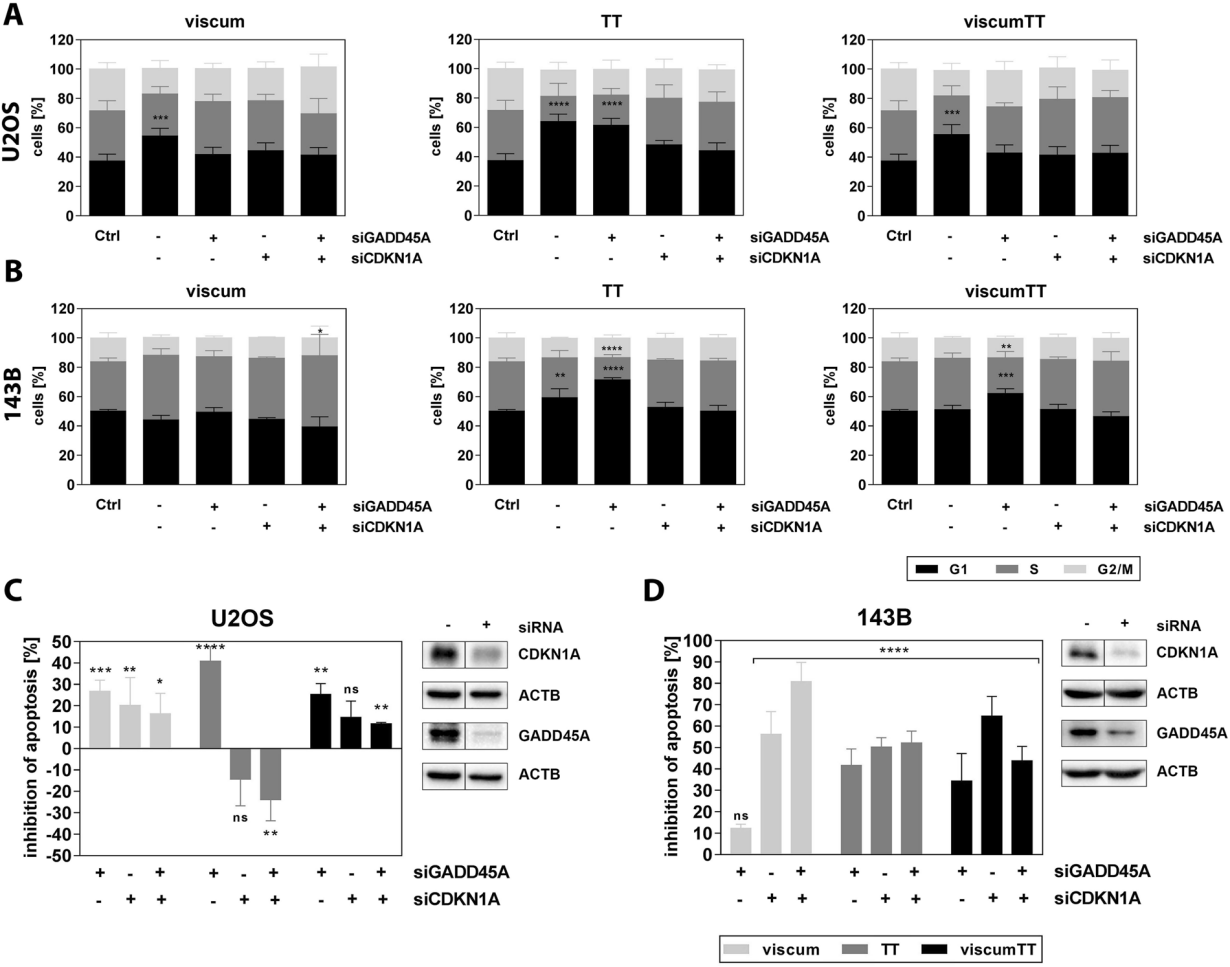
GADD45A and CDKN1A are involved in cell cycle arrest and apoptosis by viscum, TT and viscumTT. After 48 h of siRNA transfection and further 24 h of treatment with viscum, TT and viscumTT, cells were analyzed by FACS regarding cell cycle distribution U2OS (**A**) and 143B (**B**) by propidium iodide and induction of apoptosis U2OS (**C**) and 143B (**C**) by Annexin V/propidium iodide staining. Represented are the means ± SD of cells in [%] in cell cycle phase and inhibition of apoptosis [%] of at least three independent experiments and significances are indicated as *p ≤ 0.05, **p ≤ 0.01, ***p ≤ 0.001, ****p ≤ 0.0001 related to untreated control. Cropped blots evidence siRNA knock down and represent images from three independent experiments.

To further examine the role of CDKN1A and GADD45A in inhibition of cell cycle progression and induction of apoptosis, siRNA knock down experiments were performed. The viscum-induced G1 arrest (55.2%) was equally abolished when *TP53* wild type (U2OS) cells were transfected with either siGADD45A or siCDKN1A (Fig. 6A). The combination of both siRNAs reduced the number of cells in G1 phase (41.4%) but was also associated with a slight increase of cells in G2/M phase compared to single siRNAs in U2OS cells. Neither variant of siRNA had any influence on untreated U2OS cells (Figure S2). The TT-mediated G1 arrest was not prevented by knock down of *GADD45A* but attenuated by siCDKN1A and combined siRNAs (Fig. 6A). G1 arrest after viscumTT treatment was equally inhibited by all three knock down variants in these cells (Fig. 6A). In *TP53* mutant (143B) cells viscum led to a non-significant increase of cells in S phase but this effect was reduced by siGADD45A, left unchanged by siCDKN1A and enhanced by combined siRNAs. Furthermore, the TT-mediated G1 arrest increased after *GADD45A* knock down and decreased after siCDKN1A and the combination. No induction of cell cycle arrest was seen after viscumTT treatment after 24 h, whereas G1 arrest was initiated after siGADD45A (Fig. 6B). In contrast, in apoptosis experiments, siGADD45A prevented a viscum-induced effect in U2OS cells, and both other siRNA variants showed a similar inhibition in U2OS cells. Weak apoptosis was rescued by siCDKN1A and almost completely by siRNA combination in 143B cells, but not by siGADD45A. Furthermore, in *TP53* wild-type cells knock down of *CDKN1A* and both genes led to stronger induction of apoptosis after TT and viscumTT treatment. In 143B cells each siRNA variant was effective in viscum, TT and viscumTT treatment, but *CDKN1A* knock down led to the greatest inhibition of apoptosis by viscumTT. Taken together, these results illustrate differences between viscum, TT and viscumTT treatment regarding the role of GADD45A and CDKN1A in cell cycle arrest and apoptosis. Furthermore, involvement of either GADD45A or CDKN1A may be influenced by the *TP53* status of the cells.

### ViscumTT inactivates MAPK1/3 and activates MAPK8 pathway

**Figure 7:**
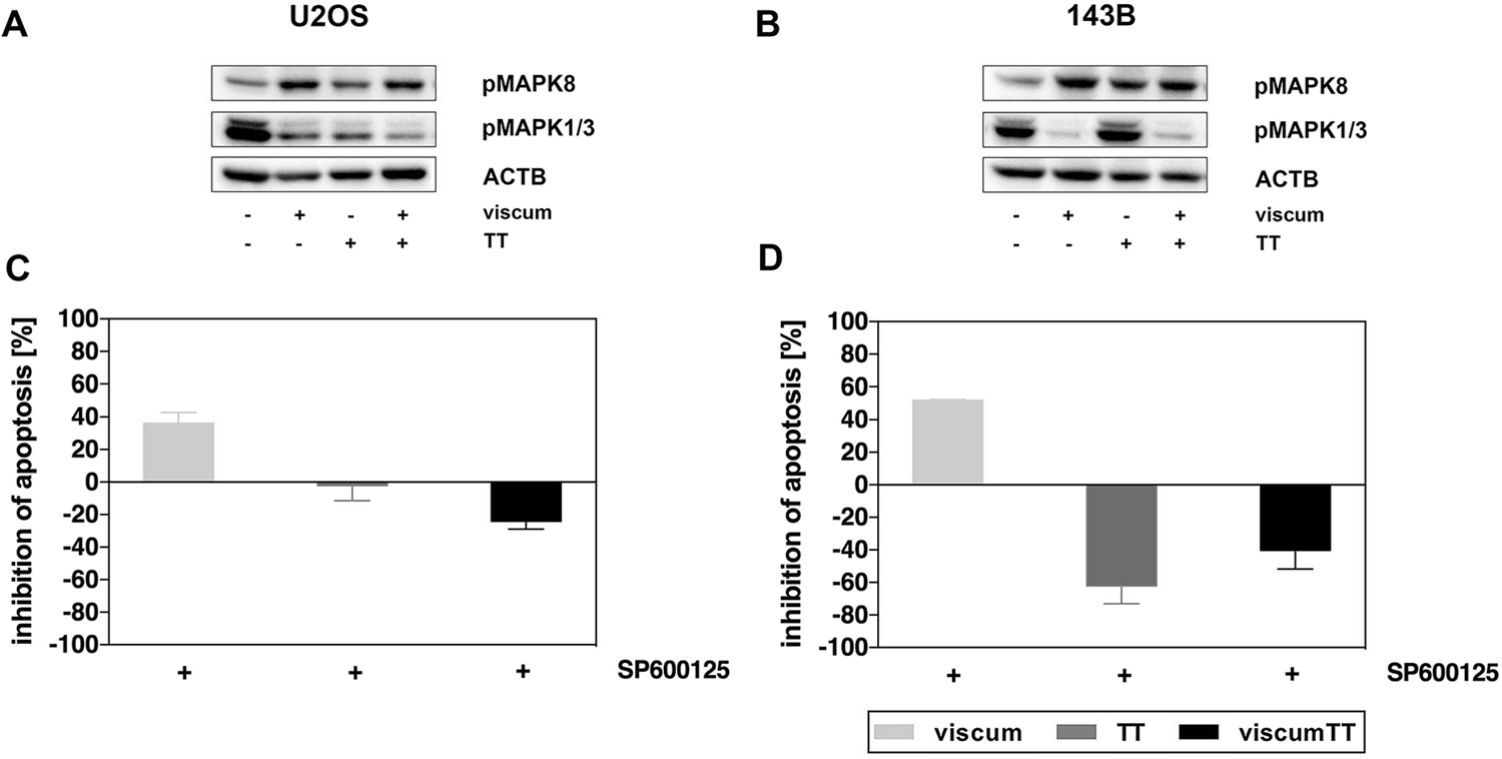
Viscum, TT and viscumTT activate stress-associated MAPK8 and inhibit survival-associated MAPK1/3. After lysis of U2OS (**A**) and 143B (**B**) cells after 24 h, phosphorylation of MAPK8 and MAPK1/3 was analyzed by western blots. ß-actin (ACTB) or GAPDH were used as loading control. Cropped blots display representative images from three independent experiments. After pre-incubation with MAPK8 inhibitor SP600125 U2OS (**C**) and 143B (**D**) cells were stained by Annexin V/PI and analyzed by FACS. Displayed are mean percentages ± SD of inhibition of apoptosis of at least three independent experiments and significances are indicated as *p ≤ 0.05, **p ≤ 0.01, ***p ≤ 0.001, ****p ≤ 0.0001 related to untreated control.

In view of the direct interaction of GADD45A and MAPK pathways, MAPK8 and MAPK1/3 activation was analyzed after viscum, TT and viscumTT treatment by western blotting. In both cell lines MAPK8 was weakly and MAPK1/3 strongly activated in control cells. After treatment with viscum and viscumTT a distinct increase in phosphorylated MAPK8 as well as a decrease in activated MAPK1/3 was seen in both cell lines (Fig. 7A,B). Additionally, after TT-treatment MAPK8 was slightly phosphorylated in both cell lines. MAPK1/3 was faintly enhanced in 143B cells. Both cell lines were further incubated with MAPK8 inhibitor SP600125. U2OS cells were sensitive to viscum (Figure S3A,B) and apoptosis was prevented up to 35% by SP600125 (Fig. 7C). As seen in an earlier study, 143B cells were nearly resistant to viscum^14^ but induction of apoptosis was reduced up to 50% (Fig. 7D). Interestingly, viscumTT-induced apoptosis in both cell lines and the TT-effect in 143B cells was significantly enhanced when preincubated with SP600125. These results show that activation of MAPK8 with simultaneous inactivation of MAPK1/3 is a molecular event in viscumTT-mediated effects.

### ViscumTT inhibits STAT3 pathway and its anti-apoptotic downstream targets

**Figure 8:**
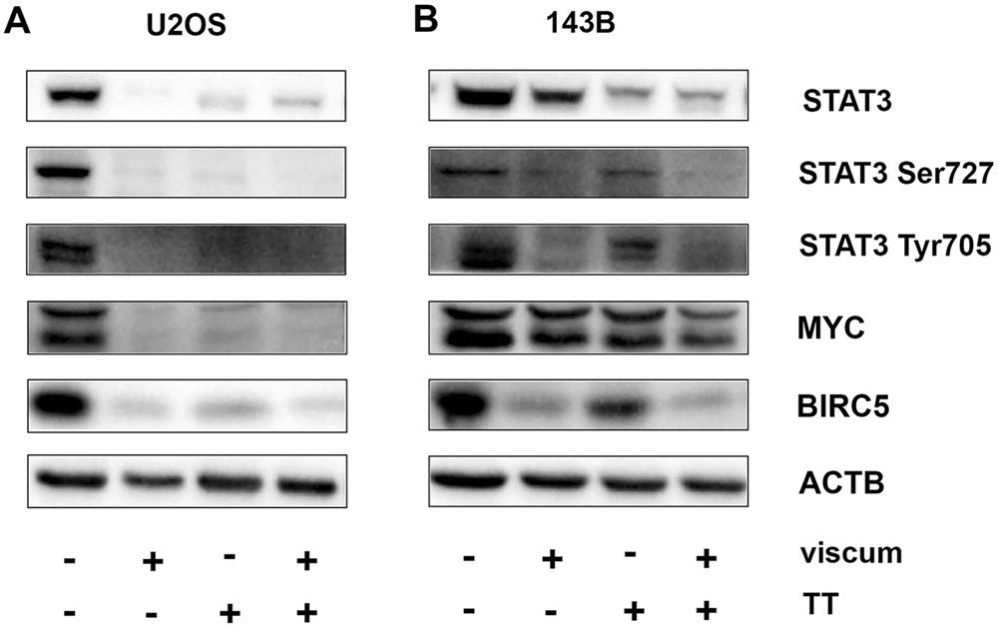
Inhibition of STAT3 pathway is involved in mechanism of action. Whole cell lysates of U2OS (**A**) and 143B (**B**) after 24 h were analyzed for total signal transducer and transcription factor 3 (STAT3) and its phosphorylation sites at Tyr705 and Ser727 as well as its direct down-stream targets baculoviral inhibitor of apoptosis repeat-containing 5 (BIRC5) and MYC-oncoprotein (MYC). ß-actin (ACTB) or GAPDH were used as loading control. Cropped blots display representative images from three independent experiments.

Phosphorylation of transcription factor STAT3 at Ser727 is associated with MAPK8 and MAPK1/3, whereas STAT3 phosphorylation at Tyr705 is mainly mediated by IL-6 and Janus kinases (JAK). In order to determine whether viscum, TT and viscumTT influence STAT3 protein levels and phosphorylation sites, cells were treated as described above and further analyzed by western blotting. Cells in both untreated controls were phosphorylated at Tyr705 and Ser727 (Fig. 8A,B). Viscum, TT and viscumTT led to dephosphorylation of Tyr705 and Ser727 with further total STAT3 degradation (Fig. 8A,B). Additionally, direct downstream targets of Tyr705 phosphorylated STAT3, such as BIRC5 and MYC-proto-oncogene (MYC) were effected. Therefore, BIRC5 was strongly down-regulated in both cell lines and MYC only in U2OS cells.

### Viscum, TT and viscumTT significantly reduce tumor volume in mice xenografts

**Figure 9:**
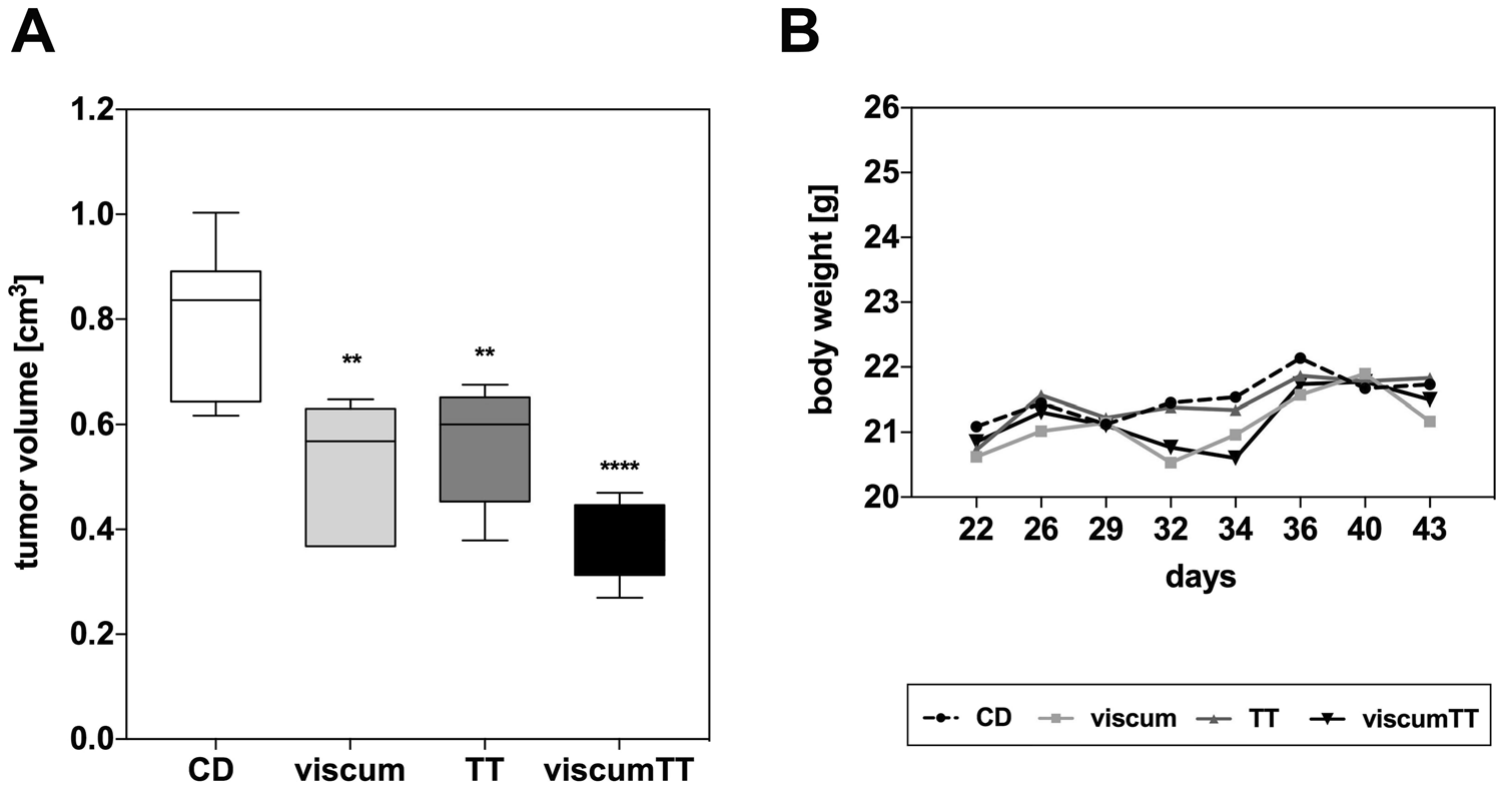
ViscumTT reduces tumor volume more effective than its single extracts. Saos-2 xenografts were intratumorally treated with 50/70/90 mg oleanolic acid (OA)/kg (OA as marker substance for TT), 0.75/1.25/1.75 µg mistletoe lectin (ML)/kg (ML as marker substance for viscum) or a combination thereof (viscumTT). Application was given twice or thrice per week in increasing concentrations whereby each concentration was administered twice. Control group received the solubilizing agent, 2-hydroxypropyl-ß-cyclodextrin (CD). Images display tumor volume (**A**), and body weight (**B**). Two-way ANOVA and Bonferroni’s multiple comparison test was used and revealed a significant group effect. Significant effects were observed between the CD control group and viscum, TT and viscumTT group and between viscumTT and viscum group (*p ≤ 0.05, **p ≤ 0.01, ***p ≤ 0.001). Indicated significances are related to CD control.

To investigate the further therapeutic potential of viscum, TT and visumTT, their efficacy was tested *in vivo*. For this purpose, Saos-2 xenografts were treated intratumorally six times for approximately three weeks. Viscum and TT led to a significant reduction of tumor volume in comparison to control group but viscumTT was more effective than the single extracts. In the viscumTT group the tumor volume was significantly reduced by approximately 50% versus the control group and a significant group effect was also revealed compared to viscum group (Fig. 9). The mice did not show any significant changes in body weight or signs of toxicity during the study which means that viscum, TT and viscumTT treatment was well tolerated.

## Discussion

The molecular mechanism of the synergistic anti-tumoral properties of viscumTT in diverse pediatric tumor entities is not fully understood^12–16,19^. The present study focused on a better understanding of the molecular function of viscumTT and its single extracts regarding cell cycle alterations and induction of apoptosis. We observed a cell cycle arrest in different phases after viscum and in G1 phase after TT treatment. Arrest in various phases of the cell cycle has also been described for commercial *Viscum album* preparations in several tumor entities^34,35^. OA-induced G1 arrest was observed in hepatocellular carcinoma cells^36^ and gallbladder cancer cells^37^ but G2/M arrest in pancreatic cancer cells^8^. Generally, cells with dysfunctional TP53 or CDKN1A lose their G1/S checkpoint and are not able to arrest in G1^38^. However, G1 arrest can be induced by various anti-cancer agents in a TP53-independent manner and is associated with induction of CDKN1A^39,40^, which was also observed in our study. Cell cycle arrest initiated by viscum either in G1 or S phase was also accompanied by up-regulation of GADD45A. In the case of DNA damage, wild-type *TP53* initiates *GADD45A* and *CDKN1A* expression resulting in G2/M or G1/S arrest, respectively^41,42^. Contrary to this, *TP53* wild-type cells (U2OS) arrested in G1 phase after viscum and viscumTT treatment through induction of *GADD45A* as has previously been described for another natural compound in cells carrying functional *TP53*^43^. Both GADD45A and CDKN1A are able to mediate their function in a TP53-dependent and -independent manner^44–47^, directly interact with^41,48^, and arrest cells either in G1/S or G2/M phase^49^. Our siRNA knockdown experiments showed joint upregulation of GADD45A and CDKN1A resulted in G1 or in S arrest (viscum, viscumTT, *TP53* mutant 143B cells) as well as in induction of apoptosis in the present study. Curcumin, another natural compound, triggered G1 arrest and apoptosis in a human adenocarcinoma cell line in a TP53-independent fashion also via simultaneous induction of GADD45A and CDKN1A^50^. A TP53-independent apoptosis induction was described several times e.g. for conventional mistletoe extracts, Korean mistletoe and recombinant mistletoe lectin but the mechanisms is not quite clear^51–53^. Additionally, in earlier studies by our group TP53 was distinctly down-regulated in a panel of tumor entities, also in osteosarcoma cells (Figure S3C)^12,14,15^. Down-regulation of TP53 might be explained by protein synthesis inhibitor function mediated by the ribosome inactivating A chain of the mistletoe lectins^52,53^. This study indicates viscumTT-induced cell cycle alterations and induction of apoptosis occur in wild-type, mutant as well as null-mutant *TP53* osteosarcoma cells. A participation of TP53 in the viscumTT mechanism of action in the wild type cells could not be excluded. TP53-independent apoptosis mechanism seems to be initiated by joint transcriptional upregulation of GADD45A and CDKN1A after viscumTT treatment. For a better understanding, the role of TP53 in the viscumTT effect has to be further investigated in the future.

MAPK8 was described as a critical target of *Viscum album* extracts^19,21,54^. MAPK8 plays a dual role in response to cellular stress stimuli which is also described for OA-induced MAPK8 and controversially discussed. On the one hand, OA induces protective autophagy via MAPK8 and mechanistic target of rapamycin (mTOR) pathway activation in various cancer cells^55,56^. On the other hand MAPK8-mediated apoptosis is reported^57^. In connection with TP53 MAPK8 forms a positive feedback loop and induces apoptosis after genotoxic stress^58^. We recently reported autophagy induction by TT and viscumTT but not by viscum in Ewing’s sarcoma cells^19^. Interestingly, the MAPK8 inhibitor SP600125 was not able to suppress apoptosis in TT and viscumTT-treated 143B and U2OS cells in this study or in Ewing’s sarcoma^19^. This phenomenon might be explain by inhibition of the OA-induced autophagy effect resulting in enhanced apoptosis^55^. Since synergistic effects of drug combinations are based on triggering different or the same targets in the same or related pathways^59^ the role of MAPK8 activation after viscumTT becomes more relevant. This observation provides pointers to the molecular mechanism of the synergistic effect of viscumTT and hypothesizes viscum’s role as autophagy inhibitor, which will have to be investigated in the future. CDKN1A might be also involved in induction of autophagy by ursolic acid, another triterpene acid^60^. Since its inhibition leads to enhanced apoptosis^61^ this supports the hypothesis of synergistic action by viscumTT.

The survival and proliferation associated MAPK1/3 pathway was distinctly inactivated by each extract in *TP53* wild-type cells, while TT did not alter phosphorylation in *TP53* mutant cells. However, MAPK1/3 activation may result in apoptosis in a TP53-independent mechanism^62^ and its inhibition led to a better effect of OA in cancer cells^63^.

In view of the combination of multiple active compounds in viscumTT, involvement of diverse pathways is very likely. The STAT3 pathway is mainly associated with survival and proliferation and often constitutively activated by phosphorylation at Tyr705 in cancer, also in osteosarcoma^64,65^. Meanwhile, other phosphorylation sites are known, Ser727 being commonly activated by MAPK1/3^66^ but also negatively regulated by MAPK8^67,68^. This was recently described for alternol, another natural compound, in osteosarcoma^69^ and for the triterpene acid ursolic acid^70^. In line with this, viscum, TT and viscumTT inactivated STAT3 at both phosphorylation sites with subsequent degradation of total STAT3 indicating involvement of that pathway in mistletoe action. Furthermore, down-regulation of the often overexpressed direct downstream targets, BIRC5 and MYC, was also observed in U2OS and 143B cells. BIRC5 down-regulation was already reported for these mistletoe extracts^12,14,15^. Depletion of STAT3 leads simultaneously to down-regulation of SKP2 and mediates cell cycle arrest^71^. SKP2 as F-box protein of the SKP1-cullin-F-box (SCF) complex catalyzes the ubiquitination of CDKN1A and CDKN1B for protein degradation in G1/S transition^72^ and may represent the key protein in STAT3 and cell cycle crosstalk. Inhibition of the STAT3/SKP2 axis by viscumTT marked a further important event in the mechanism of action.

The therapeutic effectiveness of viscum, TT and viscumTT *in vivo* was demonstrated in multiple earlier studies in diverse pediatric tumor entities^13,15,19^ and in murine melanoma^18^. In Ewing’s sarcoma and AML, viscumTT had the same effectiveness as standard chemotherapeutic drugs and showed good tolerability^12,15^. Often whole plant extracts are more effective in comparison to their single compounds^73^. In this present study we confirmed the high therapeutic potential of viscumTT, which reduced tumor volume to a greater extent than its single components. Combination therapy approaches offer the advantage of triggering multiple signaling pathways and furthermore attacking various targets which are important for fighting cancer.

## Conclusion

On the basis of our findings, we conclude that GADD45A and CDKN1A play an important role in viscumTT-mediated action in a TP53 non-essential manner. Further, we confirmed the involvement of MAPK8 and evidenced inactivation of the survival pathways MAPK1/3 and STAT3. The mechanism of action was accompanied by down-regulation of BIRC5, MYC and SKP2. Widespread triggering of pro-apoptotic targets and inhibition of survival promoting targets indicates a promising therapeutic approach for pediatric osteosarcoma.

## Materials and Methods

### Viscum album extracts

*Viscum album* extracts were prepared from apple tree (malus) mistletoe and lyophilized as described previously^16,17^ and were kindly provided by Birken AG (Niefern-Öschelbronn, Germany). In brief, intact ML I (A + B chain) in viscum extract was analyzed by ELISA^74^. Viscum was solubilized in phosphate buffered saline (PBS) resulting in a final concentration of 1 µg/mL intact ML I and <1 µg/mL viscotoxins. 2-hydroxypropyl-ß-cyclodextrin-containing TT extract was prepared in PBS to 4000 µg/mL OA and 0.35 µg/mL BA. OA (TT) and ML I (viscum) were used as marker substances for concentrations.

### Material and reagents

RPMI 1640, McCoy’s 5 A, penicillin, streptomycin, trypsin/EDTA (0.05%) and PBS were purchased from Gibco, Lifetechnologies (Darmstadt, Germany). Fetal Calf Serum (FCS) was obtained from Biochrom (Berlin, Germany). Protein inhibitors, molecular mass standards for SDS-PAGE, sodium dodecyl sulphate (SDS), dimethyl sulfoxide (DMSO) and propidium iodide (PI) were purchased from Sigma Aldrich (Munich, Germany). Beta-2 mercaptoethanol was purchased from AppliChem (Darmstadt, Germany).

### Cell culture

The human osteosarcoma cell lines 143B, Saos-2 and U2OS were obtained from American Type Culture Collection (ATCC, Manassas, VA, USA). 143B cells were cultured in RPMI 1640, Saos-2 and U2OS in McCoy’s 5 A, both supplemented with 10% heat inactivated FCS 100 U/mL penicillin and 100 µg/mL streptomycin. For the experiments, 143B and U2OS cells were seeded in 4*10^5^ and Saos-2 cells in 1*10^6^ onto 6-well plates. After 24 h attached cells were treated with viscum (ML 10 ng/mL), TT (OA 60 µg/mL) and viscumTT (5 ng/mL + 50 µg/mL) at different time points.

### Cell cycle analysis

For synchronization in G1 phase, cells were cultured in either RPMI 1640 or McCoy’s 5 A starvation medium with reduced FCS (0.04%) in 6-well microtiter plates for 24 h. Thereafter, cells were treated with viscum (ML 10 µg/mL), TT (OA 60 µg/mL) and viscumTT (ML 5 ng/mL + OA 50 µg/mL) for 3–24 h. After cells were harvested and washed with cold PBS, 1*10^6^ cells were fixed with dribs and drabs ice cold 70% ethanol under gently vortexing. Then, fixed cells were stored at −20 °C until analyses. Within two weeks, DNA fragmentation was stained with propidiumiodide (PI). Briefly, fixed cells were centrifuged and washed with cold PBS and treated with 100 µL RNAse (50 µg/mL) for 30 min at 37 °C. Finally, cells were stained with 200 µL of PI (50 µg/mL) for further 30 min on ice, in the dark. DNA content was analyzed after exclusion of cell duplexes (FL2-A against FL2-W). Cell cycle phases were evaluated with Dean Jett Fox method^75^ and FlowJo^®^ Software (FlowJo, Ashland, OR, USA).

### RNA isolation

RNA was isolated by spin columns using NucleoSpin^®^ RNA Kit (Machery&Nagel, Düren, Germany). Therefore, cells were incubated with viscum, TT and viscumTT in 6-well plates as described above. Afterwards, cells were harvested after various time points and RNA was isolated according to the manufacturer’s protocol. Concentration and purity was determined by OD 260/280 using NanoDrop™ 2000 spectrophotometer (Thermo Scientific, Dreieich, Germany). RNA integrity was checked on a denaturing agarose gel.

### RNA to cDNA transcription

cDNA was synthesized using High Capacity RNA-to-cDNA™Kit (Applied Biosystems™, Darmstadt, Germany) and 500 ng RNA of cells were transcribed according to the manufacturer’s protocol.

### RT² Profiler™ PCR Array of cell cycle genes

Alterations in 84 cell cycle genes were analyzed by RT² Profiler™ PCR Array Human Cell Cycle (Qiagen, Hilden, Germany). For this, untreated control cells and cells treated with viscum, TT and viscumTT for 24 h were investigated. Array analysis was performed with all three cell lines according to the manufacturer’s protocol. Briefly, cDNA templates were prepared in SYBR^®^ Green master mix and 25 µL of each sample were added to the SYBR^®^ Green optimized primers. Then a standard quantitative reverse transcription PCR (RT-qPCR) program was run (10 min, 95 °C; 15 s, 95 °C and 60 s, 60 °C, 40×). Array analysis was performed once for every cell line. The threshold was manually set to 1 and CT values were uploaded as an easy-to-use Excel-based file. For further analysis software from the Qiagen Analysis Center was used. Data analysis is based on the ΔΔCT method with normalization of the raw data to housekeeping genes. A fold-change greater than one indicated up-regulation. The fold-regulation is equal to the fold-change. A fold-change less than one indicates down-regulation. The fold-regulation is the negative inverse of the fold-change. Calculation of fold-regulation correlates with fold-change values in a biologically meaningful way.

### RT-qPCR validation of cell cycle genes

To confirm predominantly over-expressed *GADD45A* and *CDKN1A* and down-regulated *SKP2* in the RT² Profiler™ PCR Array, gene expression was validated by RT-qPCR on a StepOnePlus™ System in 96-well fast plates with Power SYBR^®^ Green Master Mix under standard conditions (10 min, 95 °C; 15 sec, 95 °C and 60 sec, 60 °C, 40×). The threshold was manually set to 1. A “no template control” and a “no reverse transcriptase control” were run. A RT-qPCR reaction was set up in a total volume of 20 μl containing 5 ng cDNA and 500 nM primers. Primers were predesigned and purchased from Integrated DNA Technologies (IDT, Leuven, Belgium). GAPDH*:* Hs.PT.39a.22214836, B2M: Hs.PT.58 v.18759587, GADD45A: Hs.PT.58.38974274, CDKN1A: Hs.PT.58.40874346.g, SKP2: Hs.PT.58.39891597.g.

Primer efficiencies were tested ranging between 90–100%. GAPDH or B2M levels were used as housekeeping genes and for normalization. The relative expression of genes was calculated by the ∆∆CT method: ΔΔCT = (CT(target,untreated) −CT(ref,untreated)) − (CT(target,treated) −CT(ref,treated)); fold-change = 2^^(−∆∆CT)^^76^.

### Western Blot

For protein expression cells were incubated with viscum, TT and viscumTT in T175 flasks for 3–24 h. The cells were washed twice and lysed using RIPA buffer (Carl Roth GmbH, Karlsruhe, Germany) or Lysis Buffer 17 (R&D systems, Minneapolis, MN) including proteinase inhibitor cocktail, PhoStop™ and pepstatin A. Protein concentration was determined by Bradford reagent. Cell lysates (30 μg protein/lane) were separated on SDS-PAGE, transferred to nitrocellulose membranes (Trans-Blot™ Turbo Transfer System, Bio-Rad Laboratories GmbH, Munich, Germany) and blocked with 5% BSA in 50 mM Tris-buffered saline containing 0.05% Tween-20 (TBST) for 1 h at room temperature. Blots were incubated overnight at 4 °C in TBST containing 5% BSA and primary antibody, washed twice in TBST and incubated 1 h with HRP-conjugated secondary antibodies (anti-rabbit, anti-mouse, Bio-Rad) then visualized by ECL (Thermo Scientific, Dreieich, Germany) on a Molecular Imager ChemiDoc (Bio-Rad). The following primary antibodies were used. GADD45A (#9662), CDKN1A (#2947), STAT3 (#4904), STAT3 Ser727 (#94994), STAT3 Tyr705 (#9145), pMAPK1/3 (ERK1/2, #4370), MYC (#9402) and BIRC5 (#2803) were purchased from Cell Signaling Technology (Danvers, MA, USA). SKP2 (sc-7164), pMAPK8 (sc-6254), CCNA (sc-239), CCNE (sc-247), CDK2 (sc-163), CDK4 (sc-260), TP53 (sc-73566) and GAPDH (sc25778) were purchased from Santa Cruz Biotechnology (CA, USA). ß-actin (ACTB) directly conjugated to HRP (#A3854, Sigma-Aldrich) and CCND1 (ab134175, Abcam, Cambridge, GB) were also used.

### siRNA, apoptosis detection and cell cycle analysis

For siRNA knock down experiments, U2OS and 143B cells were reverse transfected with Lipofectamine™ RNAiMAX transfection reagent (Invitrogen, Karlsruhe, Germany) and penicillin/streptomycin-free, 0.04% FCS media for 48 h. After media exchange (RPMI or McCoys, penicillin/streptomycin-free, 10% FCS) cells were incubated with viscum, TT and viscumTT for a further 24 h. *Silencer* ^TM^ Negative Control No.1 siRNA was used as non-targeting siRNA (AM4611, Thermo Scientific, Dreieich, Germany).

The following siRNAs were used:

CDKN1A:

Sense 5′-GAUGGAACUUCGACUUUGU-3′

Antisense 5′-ACAAAGUCGAAGUUCCAUC-3′

GADD45A:

Sense 5′-AAAGUCGCUACAUGGAUCAAU-3′

Antisense 5′-AUUGAUCCAUGUAGCGACUUU-3′

siRNA knock down was confirmed by western blotting. After transfection and treatment, cells were analyzed for either induction of apoptosis or cell cycle distribution. For apoptosis experiments, cells were washed twice with PBS, resuspended in 100 µL Annexin binding buffer and stained with Annexin V-APC (BD Biosciences, Franklin Lakes, NJ, USA) according to the manufacturer’s protocol. Finally, cells were stained with 1 µl PI (1 mg/mL) immediately before measuring and analyzed by flow cytometry (FACS Calibur, Becton Dickinson, Heidelberg, Germany). Data were evaluated using FlowJo^®^ Software. For cell cycle distribution, siRNA transfected cells were stained with 200 µL PI (50 µg/mL) as described above.

### Inhibitor assay

For analyzing involvement of MAPK8 activation, U2OS and 143B cells were pre-treated with 5 μM SP600125 (Sigma-Aldrich) for 1 h. DMSO was used as solvent control. Additionally, pre-treated cells were further incubated with viscum, TT and viscumTT for 24 h. After treatment, cells were washed twice with PBS and analyzed for apoptotic cells with Annexin-V/PI as described above.

### Osteosarcoma xenograft

Eight-week-old female NOD.Cg-*Prkdc*^*scid*^ *Il2rg*^*tm1Sug*^/JicTac mice were obtained from Taconic Biosciences GmbH, Cologne, Germany. The mice were housed in a pathogen-free facility under pathogen-free conditions and fed autoclaved standard diet (Sniff, Soest, Germany) with acidified drinking water *ad libitum*. Saos-2 cells (1 × 10^7^) were subcutaneously injected into the left flank and treatment started after day 21, when tumor was palpable. Viscum (0.75/1.25/1.75 μg/kg ML I), TT (50/70/90 mg/kg OA) and the combination thereof viscumTT were administered intratumorally twice a week. Each concentration was given twice. Control mice were treated with CD. Mice were monitored daily for health and symptoms of toxicity, and tumor volume was measured twice weekly. On day 43 the mice were sacrificed by cervical dislocation or sacrificed earlier if considered moribund (tumor volume >1.2 cm^3^ or >10% body weight lost). The experiments were performed in accordance with German legislation on the care and use of laboratory animals and with the United Kingdom Coordinating Committee on Cancer Research Guidelines for the Welfare of Animals in Experimental Neoplasia so as to minimize suffering. Approval for the study was obtained from the Regional Office for Health and Social Affairs (LaGeSo, approval A0452/08).

### Statistics

All *in vitro* experiments were independently performed thrice. RT² Profiler™ PCR Array analysis was performed once for each cell line and the fold-regulation cut-off was set to 2 and the p-value cut off was set to p ≤ 0.05. For each experiment, mean ± standard deviation (SD) were calculated. Two-way ANOVA and Tukey’s multiple comparison test were used for calculating significance. All significant results are indicated as *p ≤ 0.05, **p ≤ 0.01, ***p ≤ 0.001, ****p ≤ 0.001. For mice experiments, two-way ANOVA and Bonferroni’s multiple comparison test were used for determination of all significant differences between mouse xenograft groups. For synergistic induction of apoptosis (Figure S2A) the combination index (CI) was calculated by Bliss independent model: synergism (CI < 1), additive effect (CI = 1), antagonism (CI > 1)^77^. Effects and CI were calculated as follows:

$$\begin{array}{rcl}{\rm{Eab}} & = & {\rm{Ea}}+{\rm{Eb}}(1-{\rm{Ea}})={\rm{Ea}}+{\rm{Eb}}-{\rm{EaEb}}\,{\rm{where}}\,0\le {\rm{Ea}}\le 1\,{\rm{and}}\,0\le {\rm{Eb}}\le 1\\ {\rm{CI}} & = & \frac{{\rm{Ea}}+{\rm{Eb}}-{\rm{EaEb}}}{{\rm{Eab}}}\end{array}$$

Ea-Effect of substance a (viscum), Eb-effect of substance b (TT), Eab-effect of the combination of substance a and b.

## Electronic supplementary material

Supplementary information

## References

[1] Scheffler, A., Richter, C., Beffert, M., Errenst, M. and Scheer, R. *Differenzierung der Mistelinhaltsstoffe nach Zeit und Ort*. 49–76 (Hippokrates Verlag, 1996).

[2] Jung ML, Baudino S, Ribereau-Gayon G, Beck JP (1990). Characterization of cytotoxic proteins from mistletoe (Viscum album L.). Cancer Lett.

[3] Stirpe F, Sandvig K, Olsnes S, Pihl A (1982). Action of viscumin, a toxic lectin from mistletoe, on cells in culture. J Biol Chem.

[4] Schrot J, Weng A, Melzig MF (2015). Ribosome-Inactivating and Related Proteins. Toxins.

[5] Jager S, Winkler K, Pfuller U, Scheffler A (2007). Solubility studies of oleanolic acid and betulinic acid in aqueous solutions and plant extracts of Viscum album L. Planta Med.

[6] Bussing A (1996). Induction of apoptosis in human lymphocytes treated with Viscum album L. is mediated by the mistletoe lectins. Cancer Lett.

[7] Bussing A (1999). Expression of mitochondrial Apo2.7 molecules and caspase-3 activation in human lymphocytes treated with the ribosome-inhibiting mistletoe lectins and the cell membrane permeabilizing viscotoxins. Cytometry.

[8] Wei J (2013). Oleanolic acid arrests cell cycle and induces apoptosis via ROS-mediated mitochondrial depolarization and lysosomal membrane permeabilization in human pancreatic cancer cells. Journal of applied toxicology: JAT.

[9] Stein GM, Bussing A, Schietzel M (2002). Stimulation of the maturation of dendritic cells *in vitro* by a fermented mistletoe extract. Anticancer Res.

[10] Lee CH, Kim JK, Kim HY, Park SM, Lee SM (2009). Immunomodulating effects of Korean mistletoe lectin *in vitro* and *in vivo*. Int Immunopharmacol.

[11] Zhou R (2011). Inhibition of mTOR signaling by oleanolic acid contributes to its anti-tumor activity in osteosarcoma cells. Journal of orthopaedic research: official publication of the Orthopaedic Research Society.

[12] Twardziok M (2016). Multiple Active Compounds from Viscum album L. Synergistically Converge to Promote Apoptosis in Ewing Sarcoma. PLoS ONE.

[13] Stammer RM (2017). Synergistic Antitumour Properties of viscumTT in Alveolar Rhabdomyosarcoma. Journal of Immunology Research.

[14] Kleinsimon S (2017). ViscumTT induces apoptosis and alters IAP expression in osteosarcoma *in vitro* and has synergistic action when combined with different chemotherapeutic drugs. BMC Complementary and Alternative Medicine.

[15] Delebinski CI (2015). A Natural Combination Extract of Viscum album L. Containing Both Triterpene Acids and Lectins Is Highly Effective against AML *In Vivo*. PLoS ONE.

[16] Delebinski CI (2012). A new development of triterpene acid-containing extracts from Viscum album L. displays synergistic induction of apoptosis in acute lymphoblastic leukaemia. Cell Prolif.

[17] Struh CM, Jager S, Schempp CM, Scheffler A, Martin SF (2012). A novel triterpene extract from mistletoe induces rapid apoptosis in murine B16.F10 melanoma cells. Phytother Res.

[18] Struh CM (2013). Triterpenoids amplify anti-tumoral effects of mistletoe extracts on murine B16.f10 melanoma *in vivo*. PLoS One.

[19] Twardziok M (2017). Transcriptomic and proteomic insight into the effects of a defined European mistletoe extract in Ewing sarcoma cells reveals cellular stress responses. BMC Complementary and Alternative Medicine.

[20] Park YK, Do YR, Jang BC (2012). Apoptosis of K562 leukemia cells by Abnobaviscum F(R), a European mistletoe extract. Oncol Rep.

[21] Park R (2000). Activation of c-Jun N-terminal kinase 1 (JNK1) in mistletoe lectin II-induced apoptosis of human myeloleukemic U937 cells. Biochem Pharmacol.

[22] Klampfer L (2006). Signal transducers and activators of transcription (STATs): Novel targets of chemopreventive and chemotherapeutic drugs. Curr Cancer Drug Targets.

[23] Ryu K (2010). Oleanane triterpenoid CDDO-Me induces apoptosis in multidrug resistant osteosarcoma cells through inhibition of Stat3 pathway. BMC Cancer.

[24] Podlech O, Harter PN, Mittelbronn M, Pöschel S, Naumann U (2012). Fermented Mistletoe Extract as a Multimodal Antitumoral Agent in Gliomas. Evidence-based Complementary and Alternative Medicine: eCAM.

[25] Olivier M, Hollstein M, Hainaut P (2010). TP53 Mutations in Human Cancers: Origins, Consequences, and Clinical Use. Cold Spring Harbor Perspectives in Biology.

[26] Zilfou JT, Lowe SW (2009). Tumor Suppressive Functions ofp53. Cold Spring Harbor Perspectives in Biology.

[27] Miller CW (1996). Alterations ofthep53, Rb andMDM2 genes in osteosarcoms. Journal of Cancer Research and Clinical Oncology.

[28] Miyashita T, Reed JC (1995). Tumor suppressor p53 is a direct transcriptional activator of the human bax gene. Cell.

[29] Kastan MB (1992). A mammalian cell cycle checkpoint pathway utilizing p53 and GADD45 is defective in ataxia-telangiectasia. Cell.

[30] el-Deiry WS (1993). WAF1, a potential mediator of p53 tumor suppression. Cell.

[31] Schilling T (2009). Active transcription of the human FAS/CD95/TNFRSF6 gene involves the p53 family. Biochemical and Biophysical Research Communications.

[32] Lowe SW, Lin AW (2000). Apoptosis in cancer. Carcinogenesis.

[33] Otto T, Sicinski P (2017). Cell cycle proteins as promising targets in cancer therapy. Nat Rev Cancer.

[34] Harmsma M, Ummelen M, Dignef W, Tusenius KJ, Ramaekers FC (2006). Effects of mistletoe (Viscum album L.) extracts Iscador on cell cycle and survival of tumor cells. Arzneimittelforschung.

[35] Weissenstein U, Kunz M, Urech K, Regueiro U, Baumgartner S (2016). Interaction of a standardized mistletoe (Viscum album) preparation with antitumor effects of Trastuzumab *in vitro*. BMC Complementary and Alternative Medicine.

[36] Zhu Y-Y, Huang H-Y, Wu Y-L (2015). Anticancer and apoptotic activities of oleanolic acid are mediated through cell cycle arrest and disruption of mitochondrial membrane potential in HepG2 human hepatocellular carcinoma cells. Molecular Medicine Reports.

[37] Li H-F (2015). Oleanolic acid induces mitochondrial-dependent apoptosis and G0/G1 phase arrest in gallbladder cancer cells. Drug Design, Development and Therapy.

[38] Deng C, Zhang P, Harper JW, Elledge SJ, Leder P (1995). Mice lacking p21CIP1/WAF1 undergo normal development, but are defective in G1 checkpoint control. Cell.

[39] Shen G, Xu C, Chen C, Hebbar V, Kong A-NT (2006). p53-independent G1 cell cycle arrest of human colon carcinoma cells HT-29 by sulforaphane is associated with induction of p21CIP1 and inhibition of expression of cyclin D1. Cancer chemotherapy and pharmacology.

[40] Jeong J-H (2010). p53-Independent Induction of G1 Arrest and p21WAF1/CIP1 Expression by Ascofuranone, an Isoprenoid Antibiotic, through Downregulation of c-Myc. Molecular Cancer Therapeutics.

[41] Kearsey JM, Coates PJ, Prescott AR, Warbrick E, Hall PA (1995). Gadd45 is a nuclear cell cycle regulated protein which interacts with p21Cip1. Oncogene.

[42] Wang XW (1999). GADD45 induction of a G2/M cell cycle checkpoint. Proc Natl Acad Sci USA.

[43] Yoshiko S, Hoyoku N (2007). Fucoxanthin, a natural carotenoid, induces G1 arrest and GADD45 gene expression in human cancer cells. In Vivo.

[44] Zeng YX, el-Deiry WS (1996). Regulation of p21WAF1/CIP1 expression by p53-independent pathways. Oncogene.

[45] Hirose T (0000). p53-independent induction of Gadd45 by histone deacetylase inhibitor: coordinate regulation by transcription factors Oct-1 and NF-Y. Oncogene.

[46] Fornace AJ (1989). Mammalian genes coordinately regulated by growth arrest signals and DNA-damaging agents. Molecular and Cellular Biology.

[47] Rosemary Siafakas A, Richardson DR (2009). Growth arrest and DNA damage-45 alpha (GADD45α). The International Journal of Biochemistry & Cell Biology.

[48] Zhao H (2000). The central region of Gadd45 is required for its interaction with p21/WAF1. Exp Cell Res.

[49] Fan W, Richter G, Cereseto A, Beadling C, Smith KA (1999). Cytokine response gene 6 induces p21 and regulates both cell growth and arrest. Oncogene.

[50] Saha A (2010). Apoptosis of human lung cancer cells by curcumin mediated through up-regulation of “growth arrest and DNA damage inducible genes 45 and 153”. Biol Pharm Bull.

[51] Lyu SY, Choi SH, Park WB (2002). Korean mistletoe lectin-induced apoptosis in hepatocarcinoma cells is associated with inhibition of telomerase via mitochondrial controlled pathway independent of p53. Arch Pharm Res.

[52] Bussing A, Multani AS, Pathak S, Pfuller U, Schietzel M (1998). Induction of apoptosis by the N-acetyl-galactosamine-specific toxic lectin from Viscum album L. is associated with a decrease of nuclear p53 and Bcl-2 proteins and induction of telomeric associations. Cancer Lett.

[53] Hostanska K (2003). Recombinant mistletoe lectin induces p53-independent apoptosis in tumour cells and cooperates with ionising radiation. British Journal of Cancer.

[54] Kim WH, Park WB, Gao B, Jung MH (2004). Critical role of reactive oxygen species and mitochondrial membrane potential in Korean mistletoe lectin-induced apoptosis in human hepatocarcinoma cells. Mol Pharmacol.

[55] Liu J (2014). Oleanolic acid induces protective autophagy in cancer cells through the JNK and mTOR pathways. Oncol Rep.

[56] Shi Y (2016). Oleanolic acid induced autophagic cell death in hepatocellular carcinoma cells via PI3K/Akt/mTOR and ROS-dependent pathway. The Korean Journal of Physiology & Pharmacology: Official Journal of the Korean Physiological Society and the Korean Society of Pharmacology.

[57] Chen JY, Zhang L, Zhang H, Su L, Qin LP (2014). Triggering of p38 MAPK and JNK signaling is important for oleanolic acid-induced apoptosis via the mitochondrial death pathway in hypertrophic scar fibroblasts. Phytother Res.

[58] Takekawa M, Saito H (1998). A Family of Stress-Inducible GADD45-like Proteins Mediate Activation of the Stress-Responsive MTK1/MEKK4 MAPKKK. Cell.

[59] Jia J (2009). Mechanisms of drug combinations: interaction and network perspectives. Nat Rev Drug Discov.

[60] Fujiwara K (2008). Pivotal Role of the Cyclin-dependent Kinase Inhibitor p21WAF1/CIP1 in Apoptosis and Autophagy. Journal of Biological Chemistry.

[61] Zhang X (2016). p21 induction plays a dual role in anti-cancer activity of ursolic acid. Experimental Biology and Medicine.

[62] Lu Z, Xu S (2006). ERK1/2 MAP kinases in cell survival and apoptosis. IUBMB Life.

[63] Liu J (2016). ERK inhibition sensitizes cancer cells to oleanolic acid-induced apoptosis through ERK/Nrf2/ROS pathway. Tumour Biol.

[64] Chen C-L (2007). Signal transducer and activator of transcription 3 is involved in cell growth and survival of human rhabdomyosarcoma and osteosarcoma cells. BMC Cancer.

[65] Abou-Ghazal M (2008). The Incidence, Correlation with Tumor Infiltrating Inflammation, and Prognosis of p-STAT3 Expression in Human Gliomas. Clinical cancer research: an official journal of the American Association for Cancer Research.

[66] Chung J, Uchida E, Grammer TC, Blenis J (1997). STAT3 serine phosphorylation by ERK-dependent and -independent pathways negatively modulates its tyrosine phosphorylation. Molecular and Cellular Biology.

[67] Gkouveris I, Nikitakis N, Karanikou M, Rassidakis G, Sklavounou A (2016). JNK1/2 expression and modulation of STAT3 signaling in oral cancer. Oncology Letters.

[68] Lim CP, Cao X (1999). Serine phosphorylation and negative regulation of Stat3 by JNK. J Biol Chem.

[69] Zuo D (2017). Alternol, a natural compound, exerts an anti‐tumour effect on osteosarcoma by modulating of STAT3 and ROS/MAPK signalling pathways. Journal of Cellular and Molecular Medicine.

[70] Pathak AK (2007). Ursolic acid inhibits STAT3 activation pathway leading to suppression of proliferation and chemosensitization of human multiple myeloma cells. Mol Cancer Res.

[71] Wang S-T, Ho HJ, Lin J-T, Shieh J-J, Wu C-Y (2017). Simvastatin-induced cell cycle arrest through inhibition of STAT3/SKP2 axis and activation of AMPK to promote p27 and p21 accumulation in hepatocellular carcinoma cells. Cell Death & Disease.

[72] Wei Z (2013). STAT3 interacts with Skp2/p27/p21 pathway to regulate the motility and invasion of gastric cancer cells. Cellular Signalling.

[73] Ji HF, Li XJ, Zhang HY (2009). Natural products and drug discovery. Can thousands of years of ancient medical knowledge lead us to new and powerful drug combinations in the fight against cancer and dementia? EMBO Rep.

[74] Jaggy C, Musielski H, Urech K, Schaller G (1995). Quantitative determination of lectins in mistletoe preparations. Arzneimittelforschung.

[75] Fox MH (1980). A model for the computer analysis of synchronous DNA distributions obtained by flow cytometry. Cytometry.

[76] Livak KJ, Schmittgen TD (2001). Analysis of relative gene expression data using real-time quantitative PCR and the 2(−Delta Delta C(T)) Method. Methods.

[77] Foucquier J, Guedj M (2015). Analysis of drug combinations: current methodological landscape. Pharmacology Research & Perspectives.

