# Supplementary material for: GADD45A and CDKN1A are involved in apoptosis and cell cycle modulatory effects of viscumTT with further inactivation of the STAT3 pathway
Source: Sci Rep. 2018 Apr 10;8:5750. doi: 10.1038/s41598-018-24075-x (PMC5893628; doi:10.1038/s41598-018-24075-x)
Supplement: Supplementary file 1 — Supplementary information [file 41598_2018_24075_MOESM1_ESM.pdf]

Supplementary information

GADD45A and CDKN1A are involved in apoptosis and cell cycle modulatory effects of viscumTT with further inactivation of the STAT3 pathway

Susann Kleinsimon[1,2], Enya Longmuss[3], Jana Rolff[4], Sebastian Jäger[5], Angelika Eggert[1], Catharina Delebinski[1]*, Georg Seifert[1§]*

[1]Department of Pediatric Oncology/ Hematology, Otto-Heubner-Centre for Pediatric and Adolescent Medicine (OHC), Charité, Universitätsmedizin, Augustenburger Platz 1, 13353 Berlin, Germany

[2]Institute of Pharmacy, Department of Biology, Chemistry, Pharmacy, Freie Universität Berlin, Germany

[3]Institute of Molecular Bioscience, The University of Queensland, St. Lucia Qld, Australia

[4]EPO GmbH, Experimental Pharmacology & Oncology Berlin, Germany

[5]Birken AG, Streiflingsweg 11, 75223 Niefern-Öschelbronn, Germany

*These authors contributed equally to this work.

§ Address of corresponding author:
Charité – Universitätsmedizin Berlin,
Otto Heubner Centre for Pediatric and Adolescent Medicine (OHC),
Department of Pediatric Oncology/Hematology,
Augustenburger Platz 1,
13353 Berlin, Germany
Tel: +49 30 450 666 087
Fax: +49 30 450 7 566 965
Email address: georg.seifert@charite.de

Viscum, TT and viscumTT alter expression of cell cycle-associated genes

Table S1A Up- and down-regulated genes by viscum, TT and viscumTT in U2OS cells.

RefSeq Number	Gene	Description	Fold-regulation viscum
NM_000051	*ATM*	Ataxia telangiectasia mutated	-7.28
NM_001184	*ATR*	ATR Ataxia telangiectasia and Rad3 related	-2.09
NM_003600	*AURKA*	Aurora kinase A	-5.3
NM_004217	*AURKB*	Aurora kinase B	-7.28
NM_016567	*BCCIP*	BRCA2 and CDKN1A interacting protein	-2.69
NM_000633	*BCL2*	B-cell CLL/lymphoma 2	-9.37
NM_001168	*BIRC5*	Baculoviral IAP repeat containing 5	-9.05
NM_007294	*BRCA1*	Breast cancer 1, early onset	-10.83
NM_000059	*BRCA2*	Breast cancer 2, early onset	-3.19
NM_001237	*CCNA2*	Cyclin A2	-20.01
NM_031966	*CCNB1*	Cyclin B1	-5.57
NM_004701	*CCNB2*	Cyclin B2	-3.17
NM_053056	*CCND1*	Cyclin D1	-2.52
NM_001759	*CCND2*	Cyclin D2	-3.72
NM_001760	*CCND3*	Cyclin D3	-3.19
NM_001238	*CCNE1*	Cyclin E1	-8.09
NM_001761	*CCNF*	Cyclin F	-12.84
NM_004060	*CCNG1*	Cyclin G1	-3.1
NM_003903	*CDC16*	Cell division cycle 16 homolog ((Saccharomyces (S.). cerevisiae))	-2.19
NM_001255	*CDC20*	Cell division cycle 20 homolog (S. cerevisiae)	-10.89
NM_001789	*CDC25A*	Cell division cycle 25 homolog A (S. pombe)	-8.18
NM_004359	*CDC34*	Cell division cycle 34 homolog (S. cerevisiae)	-3.12
NM_001254	*CDC6*	Cell division cycle 6 homolog (S. cerevisiae)	-8.14
NM_001786	*CDK1*	Cyclin-dependent kinase 1	-5.09
NM_001798	*CDK2*	Cyclin-dependent kinase 2	-3.39
NM_000075	*CDK4*	Cyclin-dependent kinase 4	-3.73
NM_003885	*CDK5R1*	Cyclin-dependent kinase 5, regulatory subunit 1 (p35)	-5.02
NM_001799	*CDK7*	Cyclin-dependent kinase 7	3.68
NM_004936	*CDKN2B*	Cyclin-dependent kinase inhibitor 2B (p15, inhibits CDK4)	3.73
NM_005192	*CDKN3*	Cyclin-dependent kinase inhibitor 3	-3.76
NM_001826	*CKS1B*	CDC28 protein kinase regulatory subunit 1B	-3.53
NM_003592	*CUL1*	Cullin 1	-2.11
NM_003591	*CUL2*	Cullin 2	-2.06
NM_005225	*E2F1*	E2F transcription factor 1	-9.34

NM_001950	*E2F4*	E2F transcription factor 4, p107/p130-binding	-2.02
NM_013376	*GADD45A*	Growth arrest and DNA-damage-inducible, alpha	24.79
NM_016426	*GTSE1*	G-2 and S-phase expressed 1	-10.9
NM_004507	*HUS1*	HUS1 checkpoint homolog (S. Pombe)	3.26
NM_014708	*KNTC1*	Kinetochore associated 1	-2.27
NM_002266	*KPNA2*	Karyopherin alpha 2 (RAG cohort 1, importin alpha 1)	-4.09

**RefSeq Number**	**Gene**	**Description**	**Fold-regulation viscum**
NM_002358	*MAD2L1*	MAD2 mitotic arrest deficient-like 1 (yeast)	-7.17
NM_004526	*MCM2*	Minichromosome maintenance complex component 2	-5.44
NM_002388	*MCM3*	Minichromosome maintenance complex component 3	-9.69
NM_006739	*MCM5*	Minichromosome maintenance complex component 5	-4.31
NM_002392	*MDM2*	Mdm2 p53 binding protein homolog (mouse)	3.82
NM_002417	*MKI67*	Antigen identified by monoclonal antibody Ki-67	-6.21
NM_005590	*MRE11A*	MRE11 meiotic recombination 11 homolog A (S. cerevisiae)	-4.28
NM_002485	*NBN*	Nibrin	-3.96
NM_002853	*RAD1*	RAD1 homolog (S. pombe)	-2.28
NM_002875	*RAD51*	RAD51 homolog (S. cerevisiae)	-6.14
NM_002894	*RBBP8*	Retinoblastoma binding protein 8	-4.49
NM_002895	*RBL1*	Retinoblastoma-like 1 (p107)	-5.24
NM_005611	*RBL2*	Retinoblastoma-like 2 (p130)	-7.09
NM_013376	*SERTAD1*	SERTA domain containing 1	8.33
NM_005983	*SKP2*	S-phase kinase-associated protein 2 (p45)	-109.43
NM_005563	*STMN1*	Stathmin 1	-2.64
NM_007111	*TFDP1*	Transcription factor Dp-1	-18.4
NM_002046	*GAPDH*	Glyceraldehyde-3-phosphate dehydrogenase	-3.05
NM_000194	*HPRT1*	Hypoxanthine phosphoribosyltransferase 1	-3.23

**RefSeq Number**	**Gene**	**Description**	**Fold-regulation TT**
NM_003600	*AURKA*	Aurora kinase A	-3.6
NM_004217	*AURKB*	Aurora kinase B	-2.89
NM_001168	*BIRC5*	Baculoviral IAP repeat containing 5	-4.5
NM_001237	*CCNA2*	Cyclin A2	-4.33
NM_031966	*CCNB1*	Cyclin B1	-3.33
NM_004701	*CCNB2*	Cyclin B2	-3.7
NM_001761	*CCNF*	Cyclin F	-4.7
NM_001255	*CDC20*	Cell division cycle 20 homolog (S. cerevisiae)	-4.93
NM_001790	*CDC25C*	Cell division cycle 25 homolog C (S. pombe)	-3.64

NM_001786	*CDK1*	Cyclin-dependent kinase 1	-3.32
NM_000389	*CDKN1A*	Cyclin-dependent kinase inhibitor 1A (p21, Cip1)	-2.48
NM_004064	*CDKN1B*	Cyclin-dependent kinase inhibitor 1B (p27, Kip1)	2.61
NM_005192	*CDKN3*	Cyclin-dependent kinase inhibitor 3	-2.62
NM_005225	*E2F1*	E2F transcription factor 1	-2.44
NM_016426	*GTSE1*	G-2 and S-phase expressed 1	-3.33
NM_002266	*KPNA2*	Karyopherin alpha 2 (RAG cohort 1, importin alpha 1)	-2.84
NM_002358	*MAD2L1*	MAD2 mitotic arrest deficient-like 1 (yeast)	-2.27
NM_002388	*MCM3*	Minichromosome maintenance complex component 3	-2.17
NM_005914	*MCM4*	Minichromosome maintenance complex component 4	-2.5
NM_002417	*MKI67*	Antigen identified by monoclonal antibody Ki-67	-3.37
NM_002853	*RAD1*	RAD1 homolog (S. pombe)	-2.28

**RefSeq Number**	**Gene**	**Description**	**Fold-regulation TT**
NM_005983	*SKP2*	S-phase kinase-associated protein 2 (p45)	-2.87
NM_005563	*STMN1*	Stathmin 1	-2.56
NM_007111	*TFDP1*	Transcription factor Dp-1	-2.15
NM_001101	*ACTB*	Actin, beta	-2.79

**RefSeq Number**	**Gene**	**Description**	**Fold-regulation viscumTT**
NM_013366	*ANAPC2*	Anaphase promoting complex subunit 2	-2.11
NM_001184	*ATR*	ATR Ataxia telangiectasia and Rad3 related	-2.47
NM_003600	*AURKA*	Aurora kinase A	-4.23
NM_004217	*AURKB*	Aurora kinase B	-3.84
NM_016567	*BCCIP*	BRCA2 and CDKN1A interacting protein	-3.09
NM_000633	*BCL2*	B-cell CLL/lymphoma 2	-2.24
NM_001168	*BIRC5*	Baculoviral IAP repeat containing 5	-5.55
NM_007294	*BRCA1*	Breast cancer 1, early onset	-9.87
NM_000059	*BRCA2*	Breast cancer 2, early onset	-2.23
NM_001237	*CCNA2*	Cyclin A2	-11.78
NM_031966	*CCNB1*	Cyclin B1	-4.2
NM_004701	*CCNB2*	Cyclin B2	-3.11
NM_053056	*CCND1*	Cyclin D1	-2.21
NM_001759	*CCND2*	Cyclin D2	-5.57
NM_001760	*CCND3*	Cyclin D3	-3.37
NM_001238	*CCNE1*	Cyclin E1	-5.67
NM_001761	*CCNF*	Cyclin F	-8.3
NM_004060	*CCNG1*	Cyclin G1	-3.59

RefSeq Number	Gene	Description	Fold-regulation viscumTT
NM_004354	*CCNG2*	Cyclin G2	3.75
NM_003903	*CDC16*	Cell division cycle 16 homolog (S. cerevisiae)	-2.75
NM_001255	*CDC20*	Cell division cycle 20 homolog (S. cerevisiae)	-9.57
NM_001789	*CDC25A*	Cell division cycle 25 homolog A (S. pombe)	-6.15
NM_004359	*CDC34*	Cell division cycle 34 homolog (S. cerevisiae)	-3.64
NM_001254	*CDC6*	Cell division cycle 6 homolog (S. cerevisiae)	-6.35
NM_001786	*CDK1*	Cyclin-dependent kinase 1	-4.04
NM_001798	*CDK2*	Cyclin-dependent kinase 2	-4.16
NM_000075	*CDK4*	Cyclin-dependent kinase 4	-2.72
NM_003885	*CDK5R1*	Cyclin-dependent kinase 5, regulatory subunit 1 (p35)	-5.12
NM_016408	*CDK5RAP1*	CDK5 regulatory subunit associated protein 1	-2.78
NM_001799	*CDK7*	Cyclin-dependent kinase 7	3.14
NM_000389	*CDKN1A*	Cyclin-dependent kinase inhibitor 1A (p21, Cip1)	2.89
NM_004936	*CDKN2B*	Cyclin-dependent kinase inhibitor 2B (p15, inhibits CDK4)	2.93
NM_005192	*CDKN3*	Cyclin-dependent kinase inhibitor 3	-3.97
NM_001274	*CHEK1*	CHK1 checkpoint homolog (S. pombe)	-3.23
NM_007194	*CHEK2*	CHK2 checkpoint homolog (S. pombe)	-3.16
NM_001826	*CKS1B*	CDC28 protein kinase regulatory subunit 1B	-2.81
NM_003591	*CUL2*	Cullin 2	-2.22
NM_005225	*E2F1*	E2F transcription factor 1	-9.67
NM_001950	*E2F4*	E2F transcription factor 4, p107/p130-binding	-2.3
NM_013376	*GADD45A*	Growth arrest and DNA-damage-inducible, alpha	20.79
NM_016426	*GTSE1*	G-2 and S-phase expressed 1	-6.51
NM_004507	*HUS1*	HUS1 checkpoint homolog (S. Pombe)	2.71
NM_014708	*KNTC1*	Kinetochore associated 1	-2.77
NM_002266	*KPNA2*	Karyopherin alpha 2 (RAG cohort 1, importin alpha 1)	-3.22
NM_004526	*MCM2*	Minichromosome maintenance complex component 2	-4.98
NM_002388	*MCM3*	Minichromosome maintenance complex component 3	-11.55
NM_005914	*MCM4*	Minichromosome maintenance complex component 4	-7.91
NM_006739	*MCM5*	Minichromosome maintenance complex component 5	-5.75
NM_002392	*MDM2*	Mdm2 p53 binding protein homolog (mouse)	-2.71
NM_002417	*MKI67*	Antigen identified by monoclonal antibody Ki-67	-3.28
NM_005590	*MRE11A*	MRE11 meiotic recombination 11 homolog A (S. cerevisiae)	-5.76
NM_002485	*NBN*	Nibrin	-4.97

NM_002853	*RAD1*	RAD1 homolog (S. pombe)	-2.64
NM_002875	*RAD51*	RAD51 homolog (S. cerevisiae)	-5.86
NM_004584	*RAD9A*	RAD9 homolog A (S. pombe)	-2.28
NM_002894	*RBBP8*	Retinoblastoma binding protein 8	-3.62
NM_002895	*RBL1*	Retinoblastoma-like 1 (p107)	-4.97
NM_005611	*RBL2*	Retinoblastoma-like 2 (p130)	-6.94
NM_013376	*SERTAD1*	SERTA domain containing 1	6.69
NM_005983	*SKP2*	S-phase kinase-associated protein 2 (p45)	-100.51
NM_007111	*TFDP1*	Transcription factor Dp-1	-13.66
NM_006286	*TFDP2*	Transcription factor Dp-2 (E2F dimerization partner 2)	-2.06
NM_002046	*GAPDH*	Glyceraldehyde-3-phosphate dehydrogenase	-3.05
NM_000194	*HPRT1*	Hypoxanthine phosphoribosyltransferase 1	-2.5

28

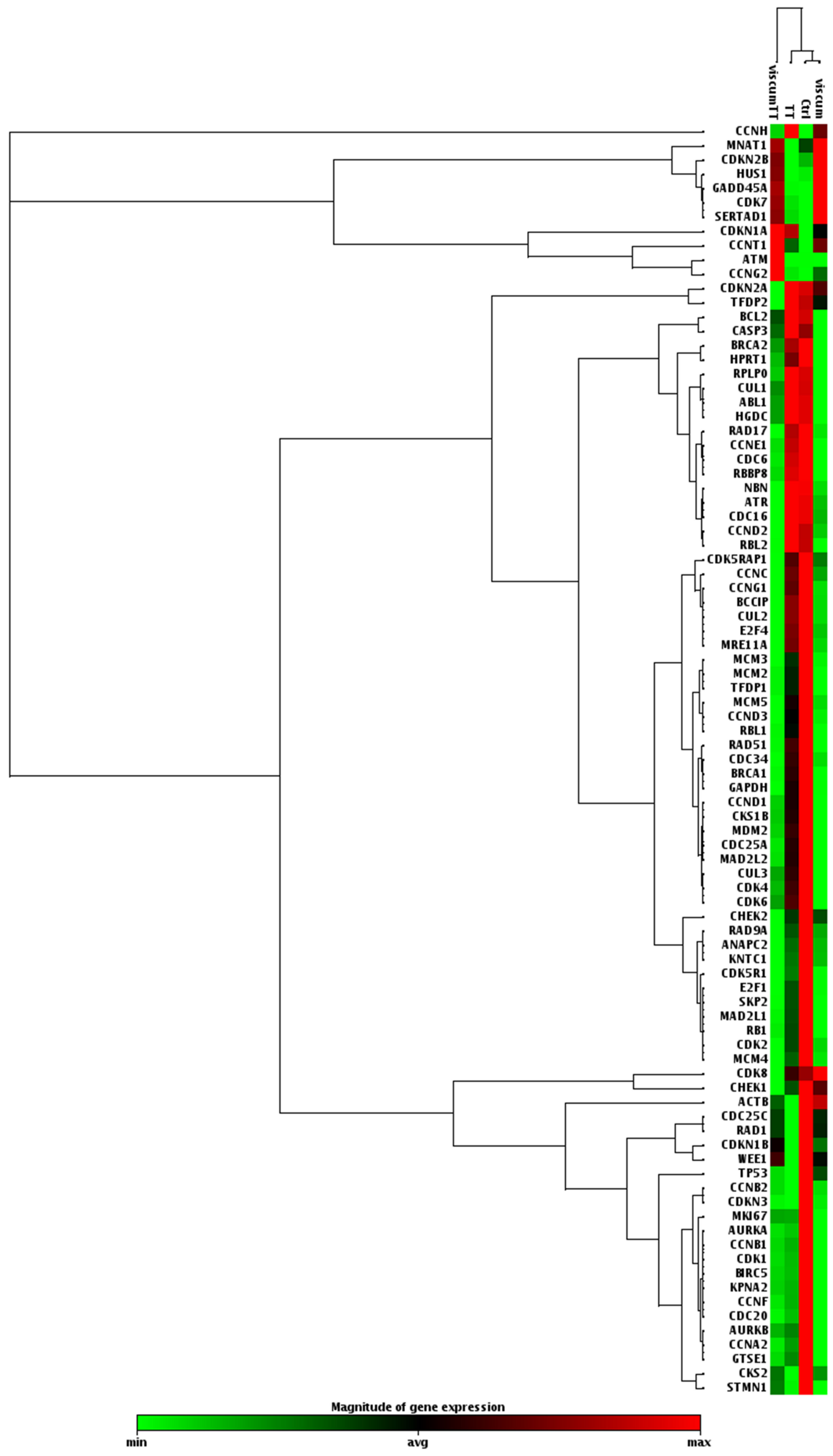

Figure S1A Clustergram of U2OS cells after viscum, TT and viscumTT treatment.

Table S1B Up- and down-regulated genes by viscum, TT and viscumTT in 143B cells.

RefSeq Number	Gene	Description	Fold-regulation viscum
NM_013366	*ANAPC2*	Anaphase promoting complex subunit 2	3.19
NM_001184	*ATR*	ATR Ataxia telangiectasia and Rad3 related	5.12
NM_007294	*BRCA1*	Breast cancer 1, early onset	-3.2
NM_000059	*BRCA2*	Breast cancer 2, early onset	2.48
NM_001237	*CCNA2*	Cyclin A2	-2.39
NM_001759	*CCND2*	Cyclin D2	2.86
NM_001238	*CCNE1*	Cyclin E1	-2.12
NM_001239	*CCNG1*	Cyclin G1	-3.38
NM_001240	*CCNH*	Cyclin H	3.96
NM_001259	*CCNT1*	Cyclin T1	2.54
NM_001254	*CDC6*	Cell division cycle 6 homolog (S. cerevisiae)	-3.53
NM_001798	*CDK2*	Cyclin-dependent kinase 2	-2.5
NM_016408	*CDK5RAP1*	CDK5 regulatory subunit associated protein 1	3.16
NM_001259	*CDK6*	Cyclin-dependent kinase 6	3.52
NM_001799	*CDK7*	Cyclin-dependent kinase 7	6.09
NM_000389	*CDKN1A*	Cyclin-dependent kinase inhibitor 1A (p21, Cip1)	5.06
NM_004064	*CDKN1B*	Cyclin-dependent kinase inhibitor 1B (p27, Kip1)	2.76
NM_005225	*E2F1*	E2F transcription factor 1	-4.2
NM_013376	*GADD45A*	Growth arrest and DNA-damage-inducible, alpha	27.99
NM_004507	*HUS1*	HUS1 checkpoint homolog (S. Pombe)	8.86
NM_006341	*MAD2L2*	MAD2 mitotic arrest deficient-like 2 (yeast)	-2.25
NM_002388	*MCM3*	Minichromosome maintenance complex component 3	-2.1
NM_005914	*MCM4*	Minichromosome maintenance complex component 4	-2.33
NM_002392	*MDM2*	Mdm2 p53 binding protein homolog (mouse)	3.82
NM_002431	*MNAT1*	Menage a trois homolog 1, cyclin H assembly factor (Xenopus laevis)	2.39
NM_002853	*RAD1*	RAD1 homolog (S. pombe)	2.55
NM_002875	*RAD51*	RAD51 homolog (S. cerevisiae)	-4.55
NM_000321	*RB1*	Retinoblastoma 1	2.26
NM_005611	*RBL2*	Retinoblastoma-like 2 (p130)	-2.74
NM_013376	*SERTAD1*	SERTA domain containing 1	8.01
NM_005983	*SKP2*	S-phase kinase-associated protein 2 (p45)	-18.07
NM_005563	*STMN1*	Stathmin 1	-4.42
NM_007111	*TFDP1*	Transcription factor Dp-1	-3.9
NM_006286	*TFDP2*	Transcription factor Dp-2 (E2F dimerization partner 2)	2.69
NM_000546	*TP53*	Tumor protein p53	2.1
NM_003390	*WEE1*	WEE1 homolog (S. pombe)	2.49

RefSeq Number	Gene	Description	Fold-regulation TT
NM_001168	*BIRC5*	Baculoviral IAP repeat containing 5	2
NM_031966	*CCNB1*	Cyclin B1	-2.2
NM_001759	*CCND2*	Cyclin D2	2.1

RefSeq Number	Gene	Description	Fold-regulation TT
NM_001239	*CCNG2*	Cyclin G2	3.05
NM_001786	*CDK1*	Cyclin-dependent kinase 1	-2.27
NM_000389	*CDKN1A*	Cyclin-dependent kinase inhibitor 1A (p21, Cip1)	6.47
NM_005192	*CDKN3*	Cyclin-dependent kinase inhibitor 3	-2.26
NM_016426	*GTSE1*	G-2 and S-phase expressed 1	-2.08
NM_002358	*MAD2L1*	MAD2 mitotic arrest deficient-like 1 (yeast)	-2.25
NM_005983	*SKP2*	S-phase kinase-associated protein 2 (p45)	-3.1
NM_007111	*TFDP1*	Transcription factor Dp-1	-2.45

RefSeq Number	Gene	Description	Fold-regulation viscumTT
NM_013366	*ANAPC2*	Anaphase promoting complex subunit 2	3.88
NM_001184	*ATR*	ATR Ataxia telangiectasia and Rad3 related	5.39
NM_016567	*BCCIP*	BRCA2 and CDKN1A interacting protein	2.19
NM_007294	*BRCA1*	Breast cancer 1, early onset	-2.85
NM_000059	*BRCA2*	Breast cancer 2, early onset	2.19
NM_001237	*CCNA2*	Cyclin A2	-2.01
NM_005190	*CCNC*	Cyclin C	2.18
NM_053056	*CCND2*	Cyclin D1	2.95
NM_001238	*CCNE1*	Cyclin E1	-2.09
NM_001239	*CCNG1*	Cyclin G1	-3.25
NM_004354	*CCNG2*	Cyclin G2	-2.17
NM_001239	*CCNH*	Cyclin H	4.38
NM_001240	*CCNT1*	Cyclin T1	3.07
NM_003903	*CDC6*	Cell division cycle 16 homolog (S. cerevisiae)	-2.79
NM_001254	*CDK2*	Cell division cycle 6 homolog (S. cerevisiae)	-2.81
NM_001798	*CDK5RAP1*	Cyclin-dependent kinase 2	3.02
NM_001259	*CDK6*	Cyclin-dependent kinase 6	3.28
NM_001799	*CDK7*	Cyclin-dependent kinase 7	5.1
NM_000389	*CDKN1A*	Cyclin-dependent kinase inhibitor 1A (p21, Cip1)	11.23
NM_004064	*CDKN1B*	Cyclin-dependent kinase inhibitor 1B (p27, Kip1)	2.46
NM_003592	*CUL1*	Cullin 1	2.15

NM_003590	*CUL3*	Cullin 3	2.17
NM_005225	*E2F1*	E2F transcription factor 1	-4.96
NM_001924	*GADD45A*	Growth arrest and DNA-damage-inducible, alpha	31.38
NM_004507	*HUS1*	HUS1 checkpoint homolog (S. Pombe)	7.69
NM_002388	*MCM3*	Minichromosome maintenance complex component 3	-2.6
NM_005914	*MCM4*	Minichromosome maintenance complex component 4	-3.07
NM_002392	*MDM2*	Mdm2 p53 binding protein homolog (mouse)	3.32
NM_002431	*MNAT1*	Menage a trois homolog 1, cyclin H assembly factor (Xenopus laevis)	2.96
NM_002853	*RAD1*	RAD1 homolog (S. pombe)	2.91

**RefSeq Number**	**Gene**	**Description**	**Fold-regulation viscumTT**
NM_005611	*RBL2*	Retinoblastoma-like 2 (p130)	-2.24
NM_013376	*SERTAD1*	SERTA domain containing 1	8.76
NM_005983	*SKP2*	S-phase kinase-associated protein 2 (p45)	-20.43
NM_005563	*STMN1*	Stathmin 1	-3.04
NM_007111	*TFDP1*	Transcription factor Dp-1	-4.45
NM_003390	*WEE1*	WEE1 homolog (S. pombe)	2.28
NM_001101	*ACTB*	Actin, beta	-2.65
NM_004048	*B2M*	Beta-2-microglobulin	2.36

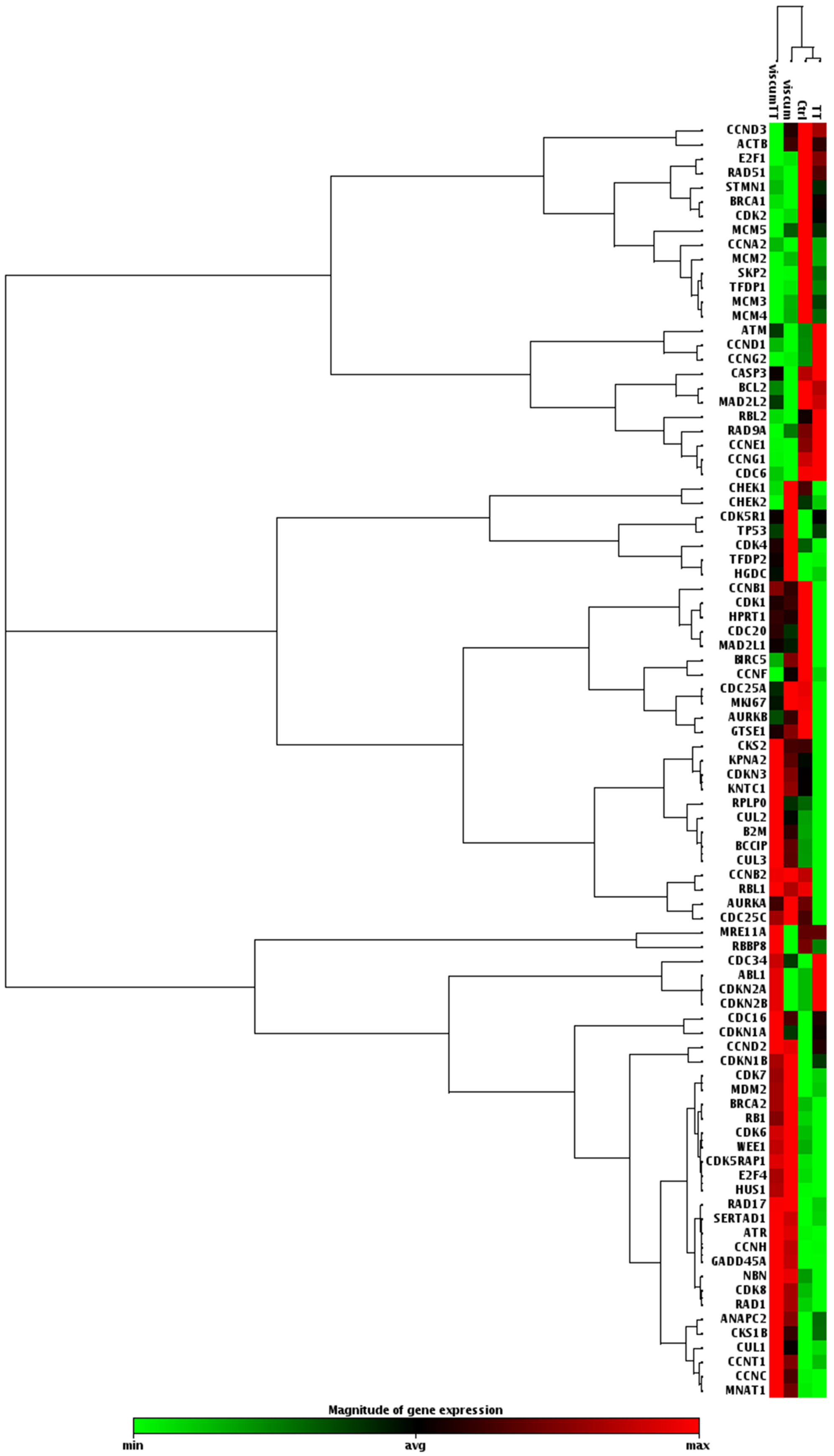

Figure S1B Clustergram of 143B cells after viscum, TT and viscumTT treatment.

Table S1C Up- and down-regulated genes by viscum, TT and viscumTT in Saos-2 cells.

RefSeq Number	Gene	Description	Fold-regulation viscum
NM_001184	*ATR*	ATR Ataxia telangiectasia and Rad3 related	2.21
NM_053056	*CCND1*	Cyclin D1	3.24
NM_004354	*CCNG2*	Cyclin G2	2.61
NM_001239	*CCNH*	Cyclin H	2.29
NM_001240	*CCNT1*	Cyclin T1	3.17
NM_001259	*CDK6*	Cyclin-dependent kinase 6	2.73
NM_001799	*CDK7*	Cyclin-dependent kinase 7	2.9
NM_004936	*CDKN2B*	Cyclin-dependent kinase inhibitor 2B (p15)	2.66
NM_001950	*E2F4*	E2F transcription factor 4	2.02
NM_001924	*GADD45A*	Growth arrest and DNA-damage-inducible, alpha	4.51
NM_004507	*HUS1*	HUS1 checkpoint homolog (S. pombe)	3.4
NM_002431	*MNAT1*	Menage a trois homolog 1, cyclin H assembly factor (Xenopus laevis)	2.37
NM_002853	*RAD1*	RAD1 homolog (S. pombe)	2.24
NM_002895	*RBL1*	Retinoblastoma-like 1 (p107)	2.11
NM_013376	*SERTAD1*	SERTA domain containing 1	4.63

RefSeq Number	Gene	Description	Fold-regulation TT
NM_003600	*AURKB*	Aurora kinase A	-2.21
NM_001168	*BIRC5*	Baculoviral IAP repeat containing 5	-3.11
NM_001237	*CCNA2*	Cyclin A2	-3.03
NM_053056	*CCND1*	Cyclin D1	2.65
NM_001759	*CCND2*	Cyclin D2	2.52
NM_001259	*CCNG2*	Cyclin G2	2.45
NM_001789	*CDC25A*	Cell division cycle 25 homolog A (S. pombe)	-2.17
NM_004359	*CDC34*	Cell division cycle 34 homolog (S. cerevisiae)	2.06
NM_001786	*CDK1*	Cyclin-dependent kinase 1	-2.88
NM_000389	*CDKN1A*	Cyclin-dependent kinase inhibitor 1A (p21, Cip1)	3.49
NM_005225	*E2F1*	E2F transcription factor 1	-2.01
NM_016426	*GTSE1*	G-2 and S-phase expressed 1	-2.11
NM_002358	*MAD2L1*	MAD2 mitotic arrest deficient-like 1 (yeast)	-2.44
NM_005914	*MCM4*	Minichromosome maintenance complex component 4	-2.35
NM_002417	*MKI67*	Antigen identified by monoclonal antibody Ki-67	-2.1
NM_000321	*RB1*	Retinoblastoma 1	2.52
NM_005983	*SKP2*	S-phase kinase-associated protein 2 (p45)	-2.46
NM_005563	*STMN1*	Stathmin 1	-2.09
NM_000546	*TP53*	Tumor protein p53	2.52

NM_001101	*ACTB*	Actin, beta	-2.54

RefSeq Number	Gene	Description	Fold-regulation viscumTT
NM_013366	*ANAPC2*	Anaphase promoting complex subunit 2	2.44
NM_001184	*ATR*	ATR Ataxia telangiectasia and Rad3 related	2.04
NM_005190	*CCNC*	Cyclin C	2.1
NM_053056	*CCND1*	Cyclin D1	6.02
NM_004354	*CCNG2*	Cyclin G2	3.66
NM_001239	*CCNH*	Cyclin H	2.2
NM_001240	*CCNT1*	Cyclin T1	2.58
NM_003903	*CDC16*	Cell division cycle 16 homolog (S. cerevisiae)	2.21
NM_004359	*CDC34*	Cell division cycle 34 homolog (S. cerevisiae)	2.39
NM_001259	*CDK6*	Cyclin-dependent kinase 6	2.16
NM_001799	*CDK7*	Cyclin-dependent kinase 7	2.46
NM_001260	*CDK8*	Cyclin-dependent kinase 8	2.27
NM_000389	*CDKN1A*	Cyclin-dependent kinase inhibitor 1A (p21, Cip1)	3.8
NM_001924	*GADD45A*	Growth arrest and DNA-damage-inducible, alpha	2.05
NM_004507	*HUS1*	HUS1 checkpoint homolog (S. Pombe)	2.56
NM_002431	*MNAT1*	Menage a trois homolog 1, cyclin H assembly factor (Xenopus laevis)	2.28
NM_013376	*SERTAD1*	SERTA domain containing 1	3.15
NM_005983	*SKP2*	S-phase kinase-associated protein 2 (p45)	-2.2

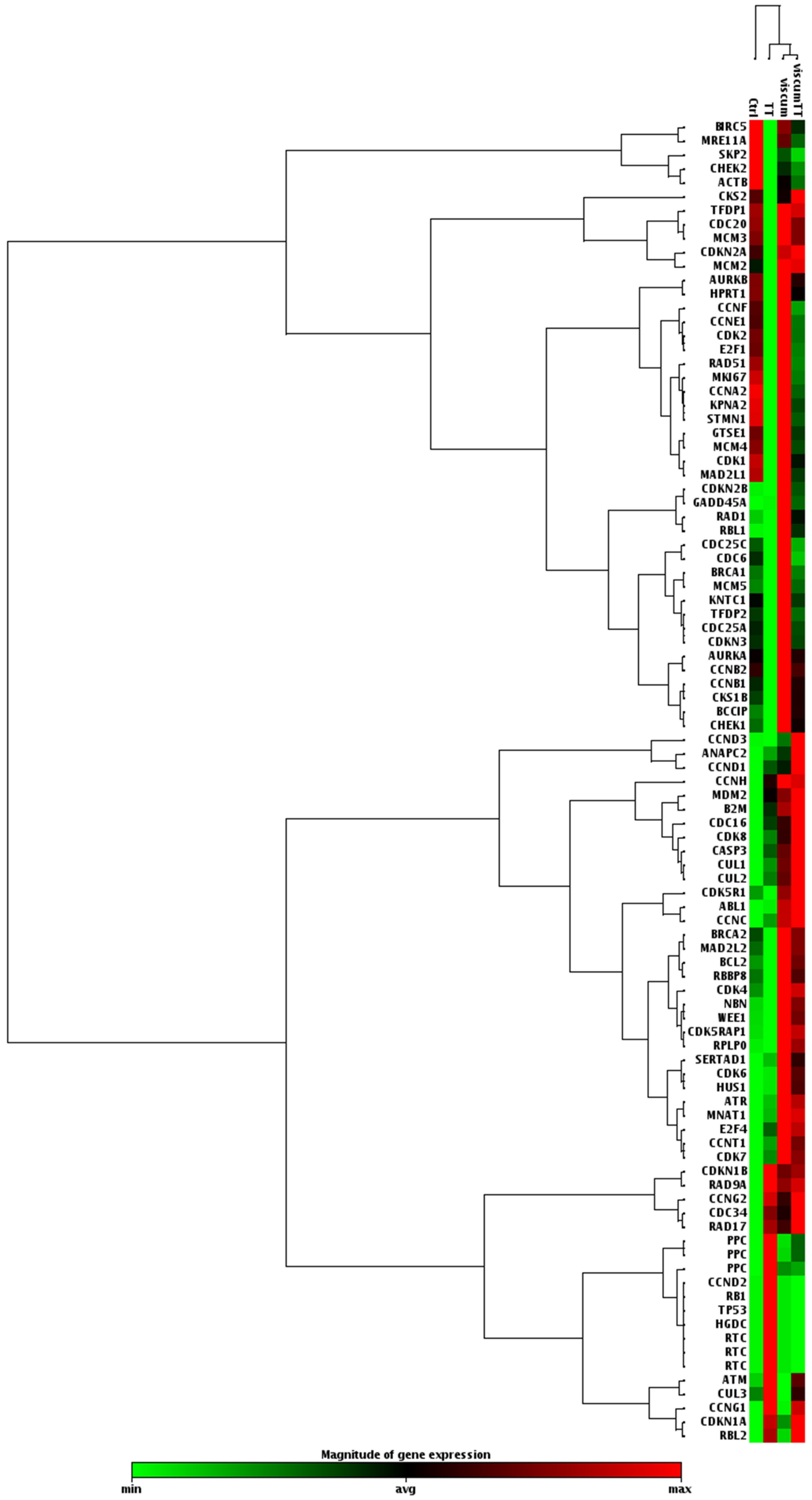

Figure S1C Clustergram of Saos-2 cells after viscum, TT and viscumTT treatment.

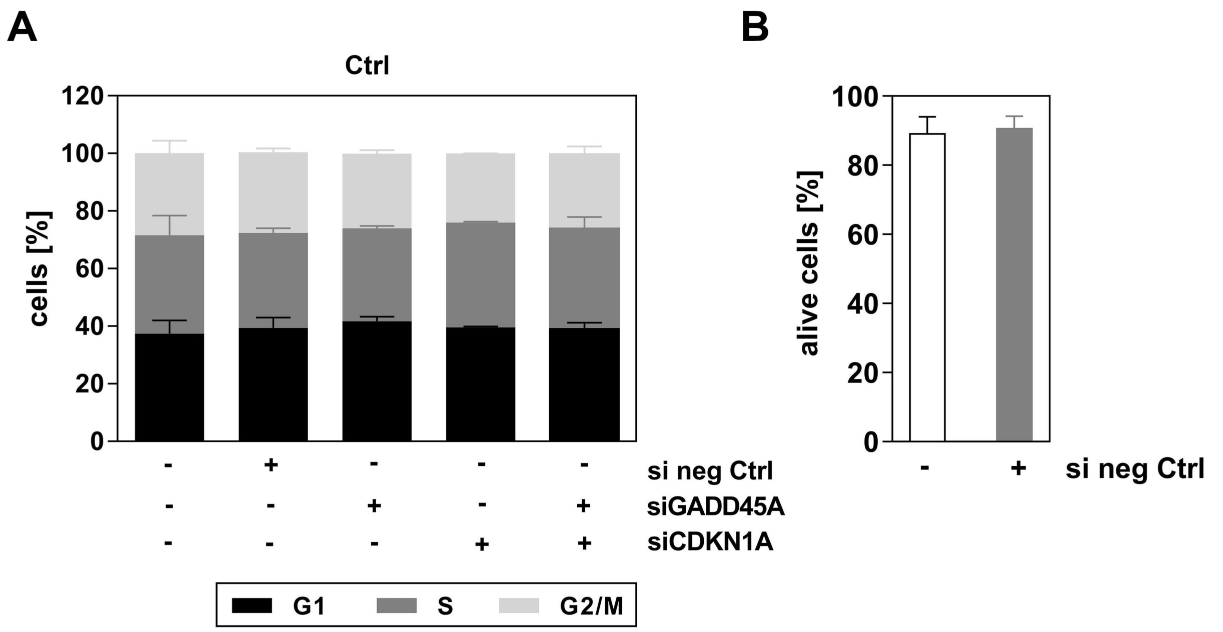

Figure S2

Control siRNA has no effect on U2OS cells

U2OS cells were reverse transfected with each variant of siRNA (siCDKN1A, siGADD45A, and the combination thereof) and non-targeting control siRNA (si neg Ctrl) for 48 h and analyzed for alterations of cell cycle distribution (A) and apoptosis induction (B) after further 24 h. Experiments were repeated thrice (n=3).

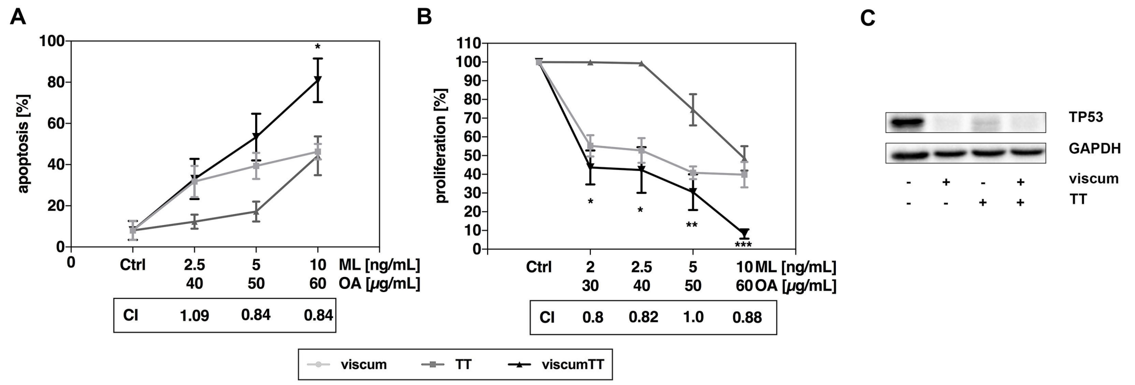

Figure S3

ViscumTT synergistically induces apoptosis, inhibits proliferation and down-regulates wild-type TP53 in U2OS cells

U2OS cells were treated for 24 h with viscum, TT and viscumTT in rising concentrations and were analyzed by Annexin/PI staining for apoptotic cells (A). Inhibition of proliferation after treatment was measured by WST-1-proliferation reagent (B) and TP53 was analyzed by western blotting after 24 h (C). Cropped blots display representative images from three independent experiments. Combination index (CI) was calculated for synergistic effects: synergism (CI < 1), additive effect (CI =1), antagonism (CI > 1). (* p≤0.05, ** p≤0.01, *** p≤0.001), (n≥3).
